# Bipolar, not tetrapolar: mating system determination in *Inonotus hispidus* through genomic and phenotypic analysis

**DOI:** 10.1007/s00253-026-13721-4

**Published:** 2026-01-29

**Authors:** Yanqi Chen, Shoujian Li, Jiao Zhang, Yuqing Jiang, Mengran Zhao, Zhihao Hou, Chenyang Huang

**Affiliations:** 1 State Key Laboratory of Efficient Utilization of Arable Land in China, Institute of Agricultural Resources and Regional Planning, Chinese Academy of Agricultural Sciences, Beijing, 100081 China; 2 Institute of Medicinal Plant Development, Chinese Academy of Medical Sciences and Peking Union Medical College, Beijing, 100193 China

**Keywords:** Sanghuang, Multinucleate mycelia, Autofluorescence, Genome sequencing, Basidiospores

## Abstract

**Abstract:**

*Inonotus hispidus* is a traditional medicinal mushroom in China with significant potential for development of health products and future foods, owing to its diverse functional components and pharmacological activities. Recent advancements in cultivation techniques, coupled with growing market demand, have expanded the production scale of *I. hispidus*. Breeding superior strains is essential for industry progress, but the absence of clamp connections in *I. hispidus* complicates mating system studies, making accurate identification of homokaryotic strains a critical step. In this study, we first confirmed the multinucleate nature of both heterokaryotic and homokaryotic mycelia, revising the traditional concepts of monokaryotic and dikaryotic mycelia in this species. Additionally, the mating type loci of *I. hispidus* were identified through genome sequencing and homologous gene BLAST analysis. Homokaryotic and heterokaryotic strains were distinguished based on sequence differences at the mating type loci between different mating types, which also allowed for differentiation of the mating types themselves. Furthermore, by combining traditional mating tests, we clearly elucidated the bipolar mating system of *I. hispidus*, refuting previous reports of a tetrapolar system. The growth rate of mycelium, its performance on a wheat grain substrate, as well as the antagonism between the homokaryotic strain and the heterokaryotic parent strain have been demonstrated to be useful for distinguishing the homokaryons. This study established a reliable method for identifying homokaryotic strains and systematically characterized the mating system of *I. hispidus* for the first time. These findings provide scientific foundation for uncovering the life cycle and presents methods for creating new germplasms.

**Key points:**

*• First confirmation of a bipolar mating system in Inonotus hispidus*

*• Single-spore isolates are multinucleate homokaryons*

*• The significant growth rate differences provide method for homokaryon identification*

**Supplementary Information:**

The online version contains supplementary material available at 10.1007/s00253-026-13721-4.

## Introduction

Sanghuang, a group of medicinal mushrooms documented in traditional Chinese medicine, has a long history of medicinal use (Bao et al. 2017; Yang et al. 2023). It was widely utilized as a health care product and ancient medicinal mushroom in Europe and East Asian countries, especially South Korea, China, and Japan (Piᶐtek 2000; Kou et al. 2021; Zhang et al. 2022; Wang et al. 2023). In 2023, the production of Chinese Sanghuang reached 400 tons, with a market size valued at 8 billion yuan (Cai 2024). Currently, *Sanghuangporus sanghuang*, *Sanghuangporus vaninii*, *Sanghuangporus baumii*, and *Inonotus hispidus* are the main species used and developed in Chinese Sanghuang industry (Yang et al. 2023). Among these, *I. hispidus* has advantages such as its wide distribution, a broad range of parasitized host tree species, and a short growth cycle (Wu and Dai 2020; Li and Bao 2022; Zhang et al. 2022; Bai et al. 2025). In recent years, the chemical composition and pharmacological effects of *I. hispidus* have been extensively studied. Recent research has demonstrated that *I. hispidus* contains various bioactive compounds, including polysaccharides, polypeptides, phenolics, steroids, flavonoids, and others, with pharmacological properties such as antitumor, immune regulation, sleep improvement, antioxidant activity, alleviation of periodontitis, and other therapeutic benefits (Kou et al. 2021; Liu et al. 2024; Wu et al. 2025; Xue et al. 2025). Furthermore, *I. hispidus* is not only a source of beneficial active ingredients with potential medicinal value but also a nutritious functional food. As a raw material, *I. hispidus* has been used to develop various products, including beverages, tea, wine, and spore powder (Xue et al. 2025).

The artificial domestication and cultivation of *I. hispidus* has been reported as early as 2007 (Cui et al. 2009). Large-scale cultivation has since been achieved in multiple provinces of China, including Hebei, Liaoning, Shaanxi, and Shandong, with standardized facility cultivation technology now established (Zhu et al. 2024; Pang et al. 2024). Notably, in the ancient Yellow River course area of Linqing City, Shandong Province, the annual production capacity reaches 8 million bags, yielding approximately 330 tons per year (Pang et al. 2024). Although there are studies on the biological activities and cultivation techniques of *I. hispidus*, only a few have focused on its genetics. In contrast to the industry's development, the seed industry has not kept pace. Currently, only Huahuang 1 and Jihuang 1, as non-main crop variety, have respectively passed the provincial approvals in Shanghai Municipality and Hebei Province, both selected from wild *I. hispidus* (Li et al. 2021b). *I. hispidus* forms fruiting bodies under temperatures of 25–30 ℃ and air moisture levels of 85%–92%. Contamination by other fungi, such as *Trichoderma* spp. and *Neurospora crassa*, remains a serious issue. Therefore, breeding superior, highly resistant strains has become increasingly important for this mushroom. Understanding the mating system and mode of reproduction is a prerequisite for effective breeding.

The sexual reproduction of basidiomycetes is complex and diverse. The process involving cellular fusion between independent strains with two distinct mating types is termed heterothallism, while sexual reproduction without a partner of a different mating type is termed homothallism. Depending on whether one or two loci control mating type and sexual reproduction, the mating system can be classified as bipolar (controlled by one mating type locus, *MAT-A*) or tetrapolar (controlled by two mating type loci, *MAT-A* and *MAT-B*) (Kües et al. 2011). Monokaryotic or homokaryotic strains serve as the fundamental materials for studying mating systems. In some basidiomycetes, the absence of clamp connections is a common morphological feature used to identify monokaryotic or homokaryotic strains (Li et al. 2020; Rebecca et al. 2021; Li et al. 2025). However, species lacking clamp connections often present challenges. For *I. hispidus*, it is widely acknowledged that clamp connections are absent in the fruiting bodies and hyphae (Davidson et al. 1942; Kühner 1950; Stalpers 1978; Cui et al. 2009). Nevertheless, one report claimed the presence of clamp connections, although the illustrated structures did not exhibit typical clamp connection morphology (Song et al. 2022). Currently, various morphological characteristics and molecular markers are employed to distinguish homokaryotic from heterokaryotic strains in fungi lacking clamp connections, as demonstrated in species such as *Phellinus sulphurascens*, *Agaricus bisporus*, and *Wolfiporia hoelen* (Lim et al. 2008; Gao et al. 2013; Li et al. 2021a). These methods provide effective approaches for studying the mating system of *I. hispidus*.

Mating types in basidiomycetes are determined by genes encoding two types of homeodomain transcription factors (*HD* genes, *MAT-A* locus) and genes encoding lipopeptide pheromones and pheromone receptors (*PR* genes, *MAT-B* locus) (Kües 2015). In general, there are two *HD* genes-*HD1* and *HD2* at least in *MAT-A* locus, and which are always linked, while the genes *MIP* (mitochondrial intermediate peptidase) and *β-fg* (beta-flanking gene) are typically conserved located in the upstream and downstream of *HD* genes. With advances in sequencing technology, genome sequencing has been extensively applied to investigate mushroom breeding (Zhu et al. 2023). The genome of *I. hispidus* has recently been sequenced (Tang et al. 2022; Zhang et al. 2022; Ding et al. 2025). Zhang et al. (2022) identified the potential mating type genes and found that the potential *MAT-A* and *MAT-B* loci were not in the same contigs, implying that *I. hispidu*s possesses a tetrapolar mating system. However, both potential *MAT-A* and *MAT-B* loci exist in species with either bipolar or tetrapolar mating systems; the key factor is which locus controls mating (Kües and Teichert 2026). Analyzing the mating type loci from a single genome to determine the mating system is insufficient. Instead, mating type locus analysis based on two genomes from compatible strains provides adequate evidence to determine the mating system, and combining this information with mating tests yields more accurate results. For example, research on the mating system of *Polyporus umbellatus* identified monokaryotic offspring with different mating types based on mating type loci, and integration with traditional mating tests clearly demonstrated a tetrapolar mating system (Li et al. 2025).

The objective of this study was to elucidate the mating system of *I. hispidus* and to distinguish between homokaryotic and heterokaryotic strains using multiple methods. These findings will aid in the screening of superior cultivars and support the industrial development of *I. hispidus*.

## Materials and methods

### Fungal materials and growth conditions

Fresh fruiting bodies were purchased from Linqing Qingyuan Original Biopharmaceutical Technology Co., Ltd., Shandong Province, China. The heterokaryotic parent strain was obtained through tissue isolation. All strains were deposited in the China Center for Mushroom Spawn Standards and Control (CCMSSC), and the heterokaryotic parent strain with the number CCMSSC05187. Identification of the heterokaryotic parent strain was based on the internal transcribed spacer (ITS) region of nuclear ribosomal DNA, with sequences deposited in GenBank under accession number PV839834. Meanwhile, this study involves several homokaryotic and heterokaryotic strains collected through spore isolation, including heterokaryotic strains: 921, 9217, and 9270; homokaryotic strains with mating type *A1*: 9210, 9215, 9228, 9229, 9231, 9260, 9274, 9284, 9299; homokaryotic strains with mating type *A2*: 924, 929, 9225, 9230, 9255, 9273, 92101, and 92106.

All strains were cultured on potato dextrose agar (PDA, Becton, Dickinson and Company, Sparks, MD, USA) medium, composed of 39 g/L potato dextrose agar, 3 g/L agar, 1 g/L KH_2_PO_4_, and 0.5 g/L MgSO_4_·7H_2_O, at 28 ℃ in the dark conditions.

### Collection of single spore isolates (SSIs)

The spore print was collected by placing the fruiting body on sterilized paper. Sterilized 200 μL pipette tips were used to collect spores from the spore prints, which were then mixed with sterile deionized water to create a spore suspension. This suspension was evenly spread in 100 μL aliquots onto PDA Petri plates (9 cm) and incubated at 28 ℃ until the spores germinated. Once visible, individual spore colonies were isolated using syringe needles and subcultured onto new PDA Petri plates at 28 ℃ in dark conditions.

### DNA preparation and genome sequencing

All strains used in this study underwent genome sequencing. The mycelia were cultured in 250 mL flasks containing 100 mL of PDB enriched medium (24 g/L potato dextrose broth, 5 g/L yeast extract, 1 g/L KH_2_PO_4_, and 0.5 g/L MgSO_4_·7H_2_O) for 10 days. They were then collected and washed three times with sterile deionized water. The mycelial samples were stored at –80 ℃ for subsequent DNA extraction and genome sequencing. Total genomic DNA was extracted using the conventional cetyltrimethylammonium bromide (CTAB) method (Doyle and Doyle 1987), and its quality was assessed with a Qubit 3.0 Fluorometer (Life Technologies, Carlsbad, CA, USA). Library preparation followed the protocols of the VAHTS® Universal Plus DNA Library Prep Kit (Vazyme, Nanjing, China), and library quality was evaluated using Qsep-400 (Bioptic Inc., Jiangsu, China) and Qubit 3.0 (Thermo Fisher Scientific, Waltham, MA, USA). Genome sequencing was performed on the DNBSEQ-T7 platform (BGI, Shenzhen, China) at Beijing Biomarker Technologies Co., Ltd. (Beijing, China).

### Mating type identification based on mating type genes

The sequences of a *Coprinopsis cinerea* HD1 protein (accession number: KAG2022959.1) and a *Gelatoporia subvermispora* beta-flanking protein (accession number: EMD41906.1) were downloaded from NCBI (https://www.ncbi.nlm.nih.gov/?hl=en). Additionally, protein sequences of *PR* genes (pheromone receptor genes) were obtained from our previous results in *P. umbellatus* (Li et al. 2025), and the MIP protein sequence was retrieved from our previous results in *W. hoelen* (Li et al. 2021a).

According to the BLAST results, two *HD1* genes were identified in the genome sequence of strain 921. Subsequently, two pairs of primers were designed based on two *HD1* genes located at the mating type *A* locus. The sequences of the primers are as follows:

- A1-HD1F1: 5′-CGGTGTCGGCGGTGTCTGA-3′
- A1-HD1R1: 5′-GCAAGTCTAACGATTTTCC-3′
- A1-HD1.2F1: 5′-GCACGAAGGAATTAGTATG-3′
- A1-HD1.2R1: 5′-CTCTCGATTCTCTGGCATG-3′

The polymerase chain reactions (PCR) were performed in a total volume of 25 µL, containing 2 µL of DNA template (100–300 ng/µL), 1 µL each of forward and reverse primers, 12.5 µL of Green *Taq* Mix (Vazyme, Nanjing, China), and 6.5 µL of ddH_2_O. The PCR conditions were as follows: initial denaturation at 95 ℃ for 3 min, followed by 35 cycles of 30 s at 95 ℃, 30 s at 53 ℃, and 1 min at 72 ℃, with a final extension at 72 ℃ for 10 min. The reactions were then stored at 4 ℃. The amplified products were separated by electrophoresis on a 2.0% agarose gel and subsequently imaged using the Gel Doc™ XR + with Image Lab™ Software version 5.2.1 (Bio-Rad, Hercules, CA, USA).

Based on the electrophoresis results, the strains that failed to amplify with two pairs of primers (A1-HD1F1/A1-HD1R1 and A1-HD1.2F1/A1-HD1.2R1), including strain 929, which was identified as a different mating type, were sent for genome sequencing again. Repeating the previous process, the *HD1* gene of strain 929 was located, and new primers were designed based on the *HD1* sequences of strain 929.

- A2-HD1F1: 5′-TGACTGAGAAACACTGGAC-3′
- A2-HD1R1: 5′-TCTCAATGCTCTCCGCCTT-3′

Different primers designed for strains 921 and 929 were used to amplify various SSIs. Electrophoresis results indicated that some strains were heterokaryotic, while the homokaryotic offspring were classified into distinct mating types.

In addition, to clarify the complete potential *MAT-A* and *MAT-B* loci, the located nodes containing *HD* and *PR* genes were annotated using Softberry online tools (www.softberry.com). Meanwhile, pheromone precursor genes were searched on the whole nodes containing *PR* genes following the previous methods (Wu et al. 2013; Kües et al. 2015).

### Mating tests

Mono-mono mating experiments were conducted using nine strains of *A1* and eight strains of *A2* mating type specificity. Blocks of two different homokaryotic strains (5 mm in diameter) were positioned 1 cm apart. Paired colonies were cultured on 6 cm PDA Petri plates for 20 days. Blocks were taken from the contact zone between the paired colonies and transferred to new 6 cm PDA Petri plates. After the mycelial colonies had grown for 10 days, a hyphal fragment from the outermost growing margin of the colony periphery was excised and transferred to new 6 cm PDA Petri plates to grow as hybrids. After 14 days culture, the mycelia in the colony margin were collected and conducted genomic DNA extraction using the CTAB method (Doyle and Doyle 1987).

### Analysis of genomic heterozygosity ratio

Clean reads were obtained by removing unmatched reads, low-quality reads, adapter contamination, and duplicate reads using fastp (Chen et al. 2018). Quality control was performed with FastQC (https://www.bioinformatics.babraham.ac.uk/projects/fastqc). The clean data (NCBI accession number: PRJNA644235) were used to estimate the heterozygosity ratio based on *k*-mer depth distribution analysis using Jellyfish 5 (Marçais and Kingsford 2011) and GenomeScope (Vurture et al. 2017; Ranallo-Benavidez et al. 2020).

### Phenotypic of heterokaryotic and homokaryotic strains

#### Growth rate on PDA

The growth rate of each isolate on a 9 cm PDA Petri plate was calculated by dividing the average colony diameter by the number of days of growth. Cultures were incubated at 28 ℃ in the dark. Colony morphology was observed when the fastest-growing strain nearly covered the plate.

### Culture characteristics on wheat grain substrate

Wheat grains were washed and then half-boiled. Afterward, the water from the wheat grains was drained. Calcium carbonate was then mixed in a 99:1 ratio. Approximately 102 ± 2 g of wheat grains were placed in a 250 mL conical flask. The conical flask was autoclaved at 121 ℃ for 30 min, then left overnight, followed by inoculation with heterokaryotic and homokaryotic strains from cultured plates. The conical flask was then incubated at 28 ℃ under dark condition.

### Antagonistic reactions

Antagonistic interaction assays were conducted by co-culturing all homokaryotic and heterokaryotic strains with heterokaryotic parent strain on 6 cm PDA Petri plates for 19 days at 28 ℃ under dark condition, after which the contact zone was observed.

### Fluorescent staining of strains and spores

The nuclear fluorescent staining of the strains was performed using a solution containing a 1:1 mixture of 50 μg/mL Hoechst 33,258 (Solarbio, Beijing Solarbio Science & Technology Co., Ltd, China) and 2.5 μg/mL calcofluor white M2R (Coolaber, Beijing Cool Shark Technology Co., Ltd, China) for 5 min in the dark (Rebecca et al. 2021). The strains were then observed using a light microscope (EVOS FL Imaging System-Life Technologies, Carlsbad, CA, USA) with an excitation wavelength of 345 nm and an emission wavelength of 455 nm.

The lipid droplets in the spores were stained with 0.1 mg/mL Nile Red (Coolaber, Beijing Cool Shark Technology Co., Ltd, China) for 5 min in the dark, then rinsed with 1 × phosphate-buffered saline (PBS) solution (pH 7.2–7.4) (Ramírez-Castrillón et al. 2021). The spores were subsequently observed using light microscopy with an excitation wavelength of 551 nm and an emission wavelength of 636 nm.

### Observation of the hymenium using scanning electron microscopy (SEM)

Fresh hymenium tissue blocks (0.15 cm in length) were cut and fixed for 2 h in 4% glutaraldehyde. Deionized water treatment, supercritical fluid drying, conductive coating, and subsequent image acquisition were performed by Wuhan Servicebio Technology Co., Ltd. (Wuhan, China). The samples were imaged using a SEM (SU8100, Hitachi, Tokyo, Japan).

### Observation of spores using transmission electron microscopy (TEM)

The spore samples were fixed for 30 min in 4% glutaraldehyde and kept at 4 ℃. Embedding, sectioning, and subsequent image acquisition were performed by Wuhan Servicebio Technology Co., Ltd, China. The samples were imaged using a TEM (HT7800, Hitachi, Tokyo, Japan).

### Statistical analyses

Growth rates are expressed as the mean ± standard deviation (SD). Significant differences were determined using Tukey test at *α* = 0.05, following one-way analysis of variance (ANOVA) performed with Jamovi 2.6.44 (https://www.jamovi.org). The growth rates of strains on PDA were generated using GraphPad Prism version 8.0.2 (GraphPad Software Inc., San Diego, CA, USA).

## Results

### Observation of the nuclear number in hyphae and the fluorescence of spores

**Fig. 1:**
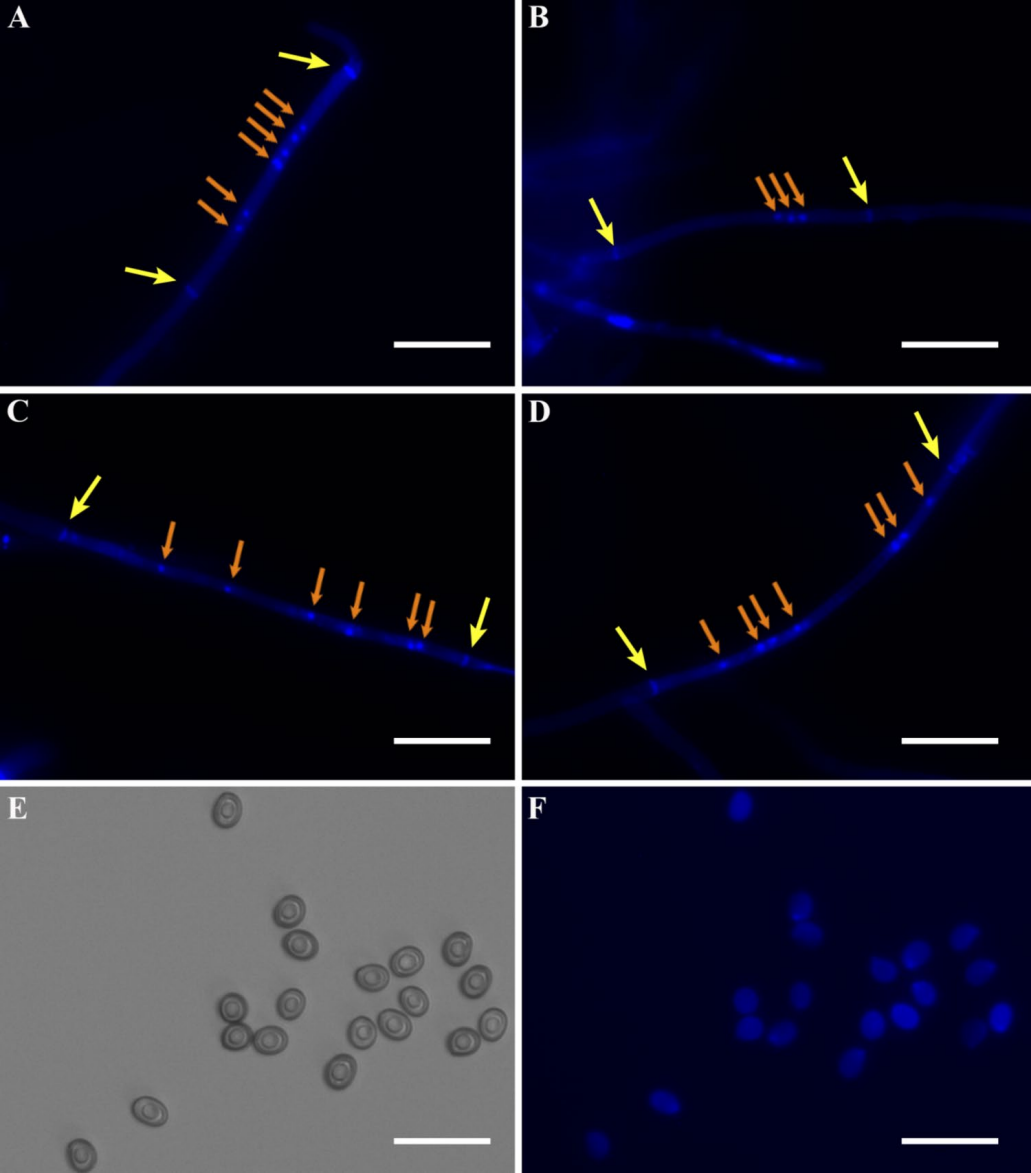
Number of nuclei in hyphae and basidiospores observed under a fluorescence microscope. (**A**) Nuclear number in hyphae of heterokaryotic parent strain. (**B**) Nuclear number in hyphae of SSIs 921. (**C**) Nuclear number in hyphae of SSIs 9228. (**D**) Nuclear number in hyphae of SSIs 92101. (**E**) Basidiospores under the microscope. (**F**) Autofluorescence phenomenon of basidiospores. The orange arrows indicate the nuclei, and the yellow arrows indicate the septa of the hyphal cell. Bars = 50 μm

The hyphae of heterokaryotic parent strain and three SSIs strains of 921, 9228, and 92101 were stained, and the number of nuclei was observed under a fluorescence microscope (Fig. 1). The hyphae of the heterokaryotic parent strain were multinucleate, exhibiting either two or three closely arranged nuclei, as well as dispersed nuclear distribution within the hyphal cells. No clamp connections were observed in the hyphae of the heterokaryotic parent strain isolated from a fresh fruiting body (Fig. 1A). Similarly, the hyphae of the three SSIs strains were also multinucleate, with nuclei arranged either in tight clusters or irregularly dispersed (Fig. 1B, C and D). Interestingly, the basidiospores of *I. hispidus* exhibited strong autofluorescence (Fig. 1F), which differs from the majority of mushrooms and precludes fluorescence-based observation of nuclear quantity.

### Mating type discrimination based on mating type loci

**Fig. 2:**
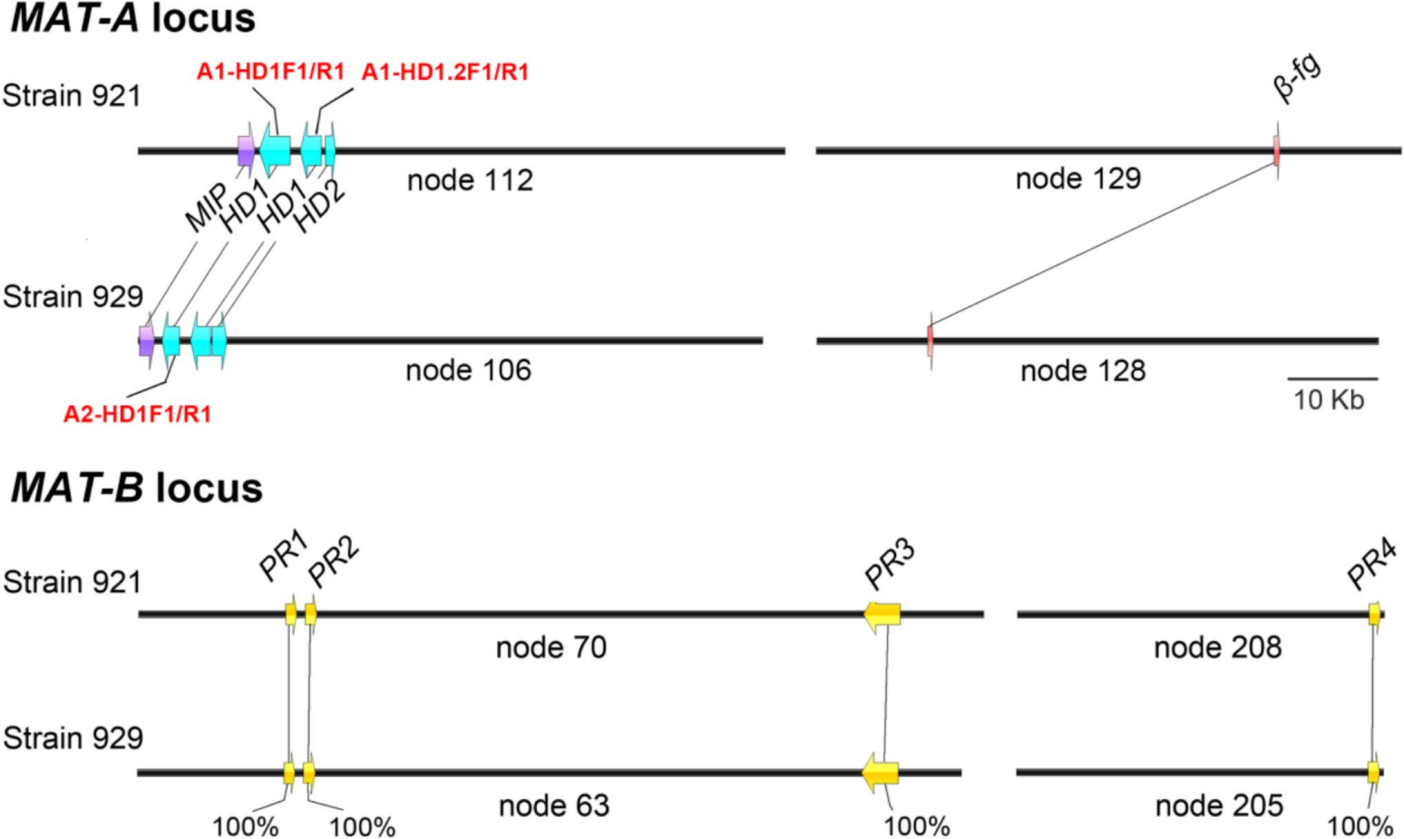
Potential mating type loci of *I. hispidus*. The potential *MAT-A* locus includes one pair of *HD1*/*HD2* and a single *HD1*, the potential *MAT-B* locus includes four *PR* genes. Purple arrows represent *MIP*; blue arrows represent *HD*; red arrows represent *β-fg*; yellow arrows represent *PR*. Bars = 10 Kb

According to the reference sequences of *HD1*, *MIP*, *β-fg*, and *PR*, the corresponding genes were identified using the tBLASTn command in the genomes of strains 921 and 929 (Fig. 2). Additionally, homologous prediction was performed to identify mating type genes using Softberry online tools, with *Serpula lacrymans* and *Rhodonia placenta* (*Postia placenta*) as reference species. In total, three *HD* genes were located, including one pair of *HD1*/*HD2* genes oriented in opposite directions and a single *HD1* gene (potential *MAT-A* locus). The *MIP* gene was also identified, while the *β-fg* gene was located on a different contigs. Furthermore, four pheromone receptor genes as potential *PR* genes were found on two separate contigs (potential *MAT-B* locus), while no pheromone precursor gene was found. The number and arrangement of genes in the potential mating type loci *MAT-A* and *MAT-B* were similar in strains 921 and 929. The *HD* genes exhibited significant divergence, 25.65% nucleotide identity of *HD1*, 79.74% nucleotide identity of *HD1.2* and 50.76% nucleotide identity of *HD2* were discovered, whereas the *PR* genes were identical with 100% similarity (Supplementary Fig. S1), indicating that mating in *I. hispidus* was probably determined by the *HD* genes, while the *PR* genes do not control mating. These findings suggest the possible bipolar mating system of *I. hispidus*.

**Fig. 3:**
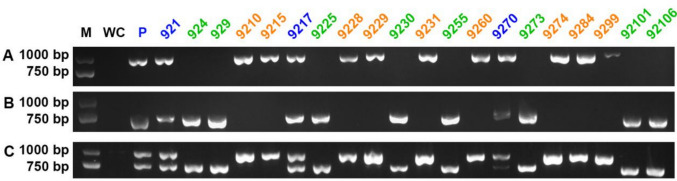
Identification of mating types based on the analysis of mating type loci. (**A**) Strains that successfully amplified with primers A1-HD1F1/R1 were designated as mating type *A1*. (**B**) Strains that successfully amplified with primers A2-HDF1/R1 were designated as mating type *A2*. (**C**) Strains that only successfully amplified with primers A1-HD1F1/R1 were designated as mating type *A1*; conversely, those that only amplified with primers A2-HDF1/R1 were designated as mating type *A2*. Strains that successfully amplified with both primers A1-HD1F1/R1 and A2-HDF1/R1 were identified as heterokaryotic strains. M indicates the 2-kb size marker. WC indicates the water control. P indicates the heterokaryotic parent strain. Blue labels indicate heterokaryotic strains; orange labels indicate homokaryotic strains with mating type *A1*; green labels indicate homokaryotic strains with mating type *A2*

In the beginning, two pairs of primers were designed based on the sequences of two *HD1* genes from strain 921. These primers were used to amplify various SSIs. Both primer pairs produced consistent results, indicating the accuracy of the located mating type genes (Supplementary Fig. S2). However, amplification failed for strain 929, which was subsequently subjected to genome sequencing (Supplementary Fig. S2). Further analysis revealed the *MAT-A* locus of the two strains, and a new pair of primers was designed based on strain 929. Clear amplified bands confirmed the high quality of the DNA templates. Based on electrophoresis results, the SSIs were classified into two distinct types (Fig. 3). Strains 9210, 9215, 9228, 9229, 9231, 9260, 9074, 9284, and 9299 were designated as mating type *A1* (Fig. 3A), while strains 924, 929, 9225, 9230, 9255, 9273, 92101, and 92106 were classified as mating type *A2* (Fig. 3B). Additionally, strains 921, 9217, and 9270 were identified as heterokaryotic strains (Fig. 3C). Furthermore, by combining the two pairs of primers into a single PCR system, heterokaryotic and homokaryotic strains with different mating types can be more easily distinguished (Fig. 3C), greatly reducing the workload.

### Validation of mating

**Fig. 4:**
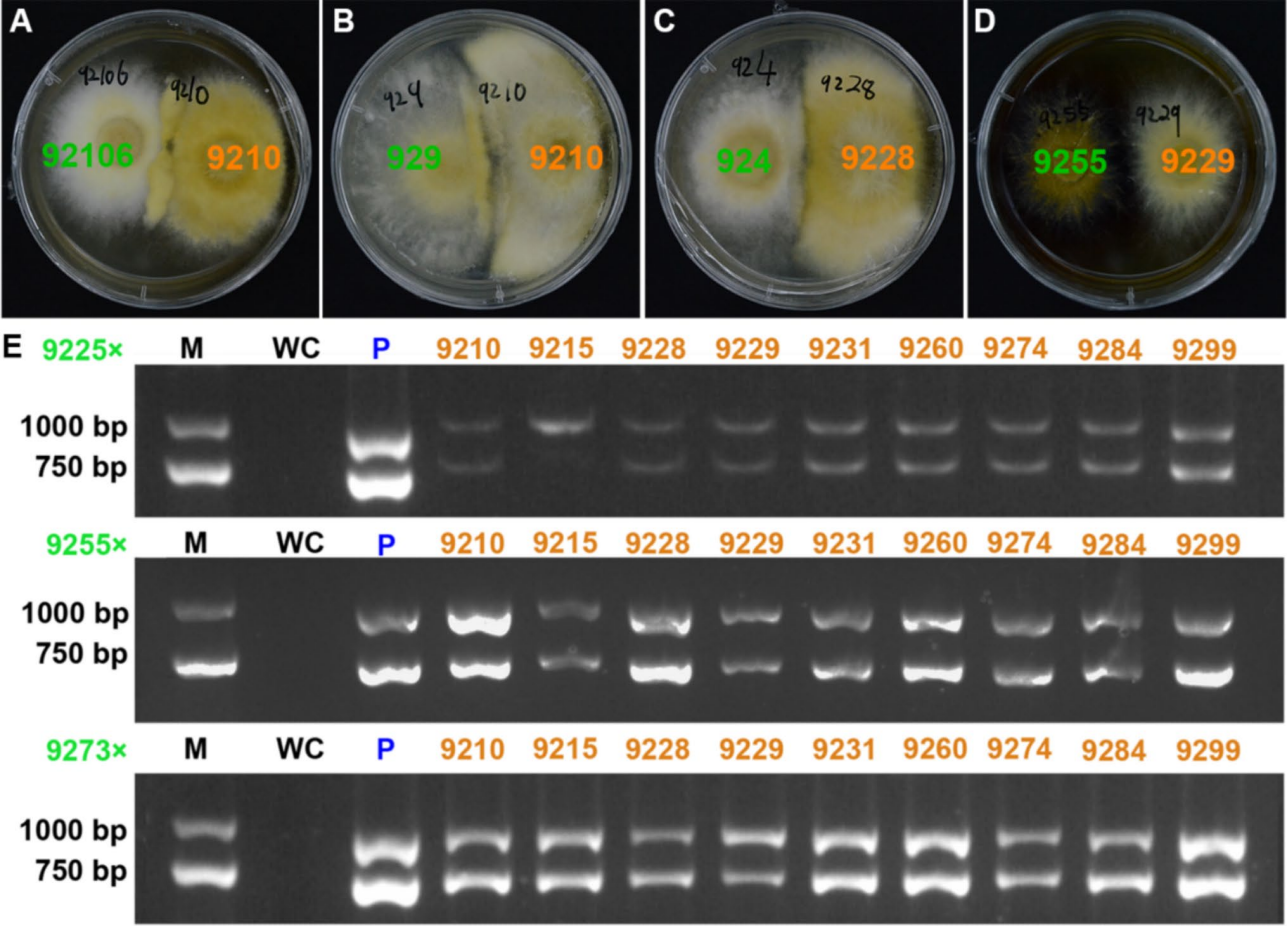
Mating reactions of SSIs. (**A**) Aerial type. (**B**) Gully type. (**C**) Contact type. (**D**) No phenomenon. (**E**) Validation of mating based on the two primers designed on mating type *A1* and *A2*. Double bands indicates successful mating, single bands indicate failed mating. M indicates the 2-kb size marker. WC indicates the water control. P indicates the heterokaryotic parent strain. Blue labels indicate heterokaryotic strains; orange labels indicate homokaryotic strains with mating type *A1*; green labels indicate homokaryotic strains with mating type *A2*

Based on the results obtained from the analysis of mating type loci, a total of 17 strains were selected for mating tests. In the mono-mono pairing tests, a total of 72 mating combinations were formed (Supplementary Fig. S3). Among them, four different reactions were observed in the contacted regions, including aerial, gully, contact and no phenomenon (Fig. 4). The mating results indicated that the gully type was the one with the highest proportion (38.89%), with a total of 28 combinations (Supplementary Fig. S3). Morphologically, white or yellow aerial mycelia are produced at the mycelial junctions. The mycelia do not interweave and there were gullies in the middle. On the reverse side, the mycelia were dense and there were either gullies or pigment accumulations in the middle. There were 22 combinations (30.56%) where obvious and dense white or yellow aerial mycelial masses were produced at the mycelial junctions (Supplementary Fig. S3). When the mating strains were the aerial types 9228 and 9225, mating with other strains may show contact types, presenting a phenomenon similar to antagonism and there were 17 combinations (23.61%). Moreover, there were 5 combinations showing no phenomenon with a proportion of 6.94%.

Three *A2* strains were selected for mating verification with 9 *A1* strains, including the aerial type 9225, the adherent type 9255 and 9273. Among the 27 mating combinations, four distinct mating phenotypes were observed. After purification and culturing of the intermediate mycelium, all mating combinations except for 9225 × 9215 produced round colony and vigorous aerial hyphae. PCR analysis was conducted to confirm successful mating (Fig. 4E). Only the *A1* locus was detected in the contact type mating combination 9225 × 9215, indicating a lack of successful mating. The aerial type, gully type, and no phenomenon type combinations achieved a 100% mating success rate, whereas the contact type mating may involve non-mating scenarios.

### Differentiating homokaryotic and heterokaryotic strains by comparing phenotypes

#### Colony morphology and growth rates of homokaryotic and heterokaryotic strains on PDA

**Fig. 5:**
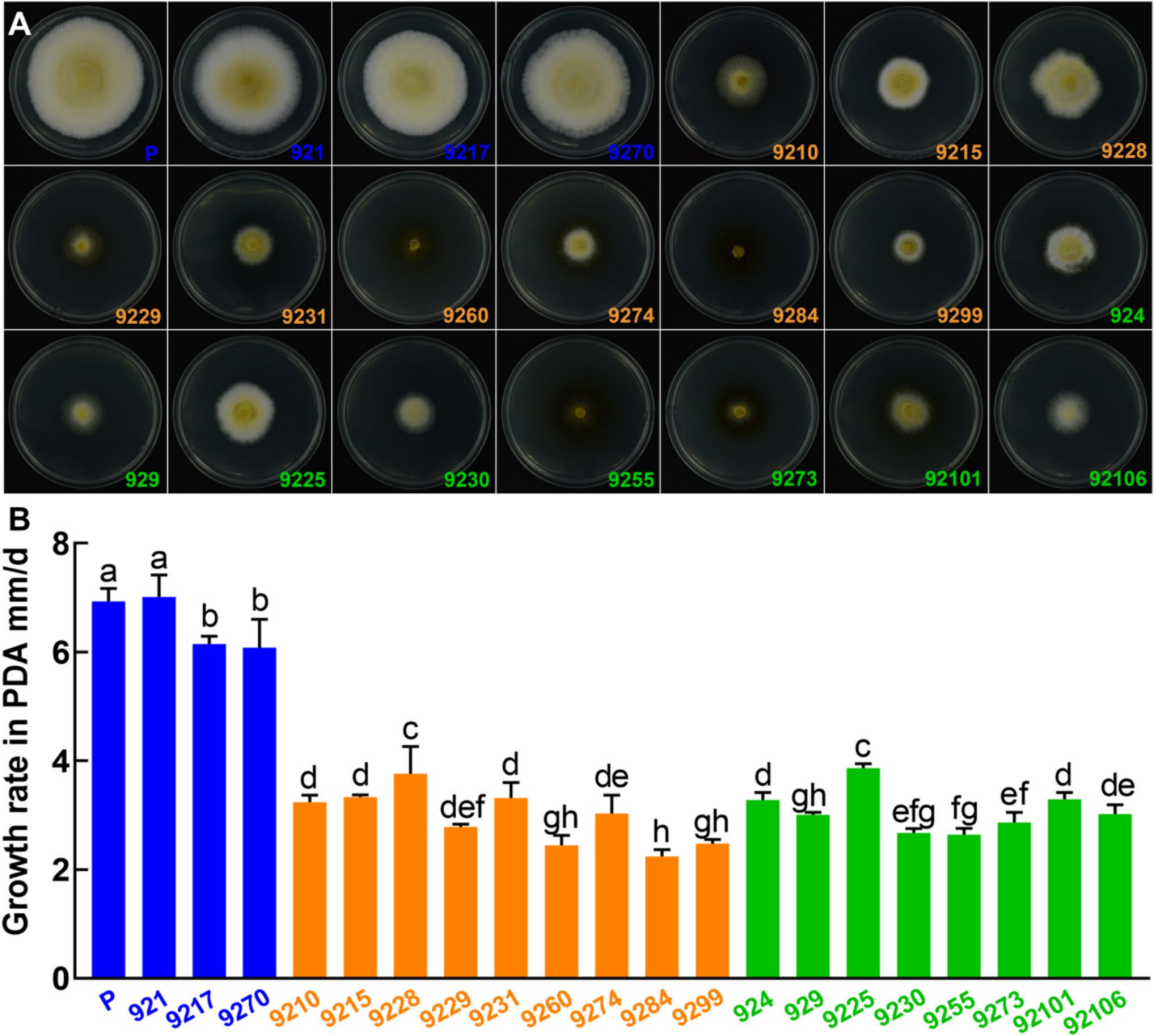
Colony morphology and growth rates of heterokaryotic and homokaryotic strains on PDA medium. (**A**) Colonies of various heterokaryotic and homokaryotic strains cultured on PDA medium. (**B**) Growth rates of different heterokaryotic and homokaryotic strains. Different letters above the bars denote significant differences at *P* < 0.05. Bars mean SD (*n* = 4). P indicates the heterokaryotic parent strain. Blue labels indicate heterokaryotic strains; orange labels indicate homokaryotic strains with mating type *A1*; green labels indicate homokaryotic strains with mating type *A2*

The 21 strains involved in the comparison of colony morphology and growth rate included the heterokaryotic parent strain, three heterokaryotic strains, and 17 homokaryotic strains (eight with mating type *A2* and nine with mating type *A1*) derived from the heterokaryotic parent strain (Fig. 5). After 10 days of culture, the heterokaryotic parent strain had nearly fully covered the plate. All heterokaryons exhibited faint yellow coloration and dense aerial mycelia, similar to the heterokaryotic parent strain (Fig. 5A). The majority of the homokaryotic strains, both *A1* and *A2* types (13 out of 17), had fewer aerial mycelia and displayed a creeping growth pattern on the media, showing distinct morphological differences. Other homokaryotic strains, such as 9215, 9228, 924, and 9225, had aerial mycelia resembling the heterokaryotic parent strain, but their growth was slower. Therefore, homokaryotic strains differ markedly from heterokaryotic strains in colony morphology and growth rate. Homokaryotic strains can be classified into two types, an aerial type and a creeping type, based on the density of aerial mycelia.

There was a significant difference in growth rates between the heterokaryotic and homokaryotic strains (Fig. 5B). The mycelial growth rate of the heterokaryotic parent strain was significantly higher than that of the other 19 strains, except for strain 921. Additionally, four heterokaryotic strains grew significantly faster than the homokaryotic strains (*P* < 0.05). Among the homokaryotic strains, there was also a significant difference in growth rates. The mycelial growth rates of homokaryotic strains *A1* and *A2* ranged from 2.24 to 3.87 mm/d. Except for strains 9225 and 9228, fifteen homokaryotic strains exhibited growth rates less than half that of the parental strain. Strains 9228 and 9225 corresponded to *A1* and *A2*, respectively, and their mycelial growth rates were significantly higher than those of other homokaryotic strains. When the mycelial growth rate is below 5 mm/d, the strain may be homokaryotic; however, the two homokaryotic strains cannot be distinguished based on the aerial and creeping morphology of the mycelium.

### Growth performance of homokaryotic and heterokaryotic strains on wheat grains substrate

**Fig. 6:**
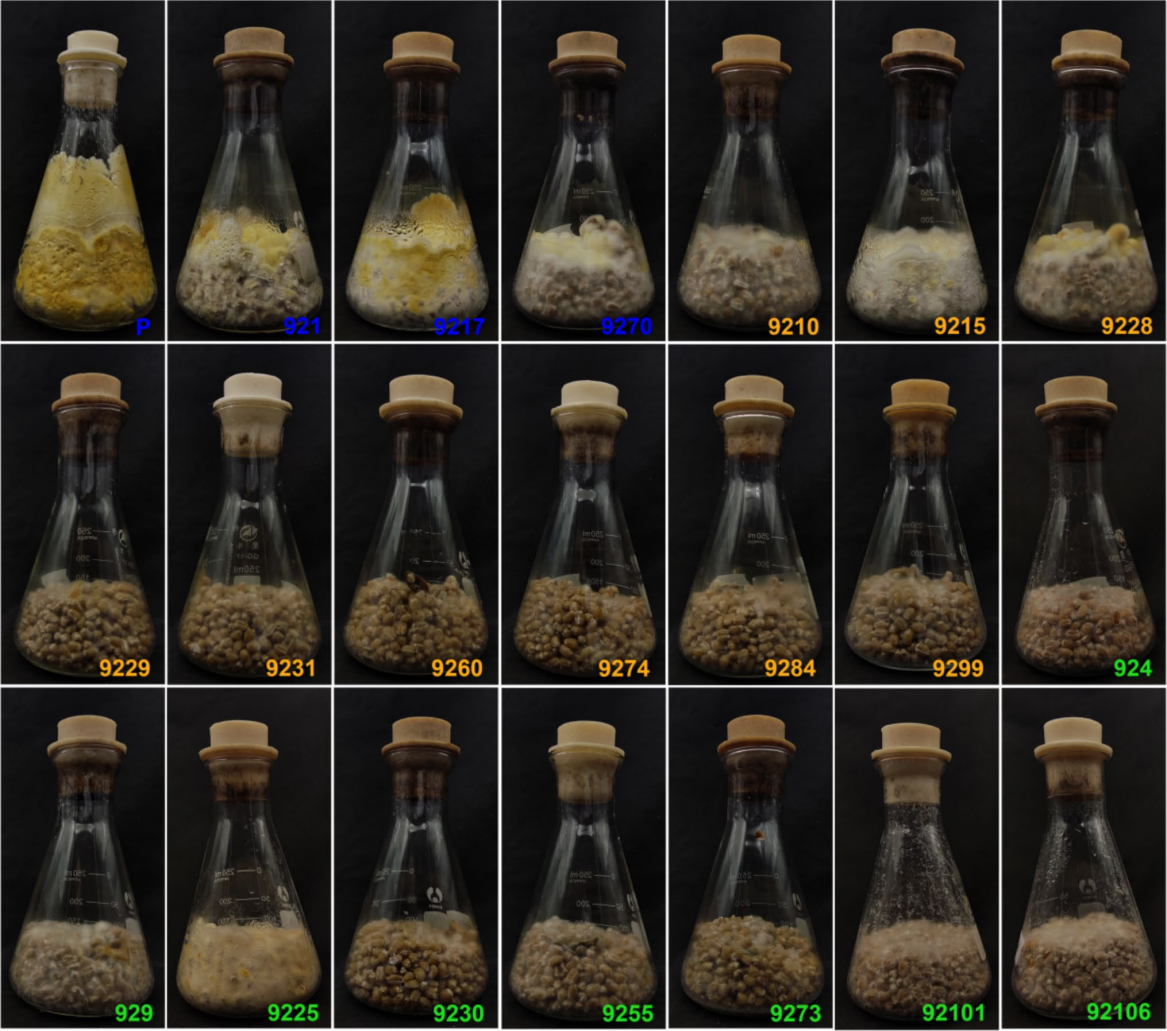
Growth performance of different homokaryotic and heterokaryotic strains on wheat grains. P indicates the heterokaryotic parent strain. Blue labels indicate heterokaryotic strains; orange labels indicate homokaryotic strains with mating type *A1*; green labels indicate homokaryotic strains with mating type *A2*

In the cultivation of edible and medicinal mushroom, wheat grains are commonly used as substrates for preparing original or cultivation strains. The growth of 21 strains on wheat grains is shown in Fig. 6. During the same culture period, the heterokaryotic parent strain exhibited not only dense aerial mycelium but also a strong ability to climb the walls of the wheat grains. In contrast, the heterokaryons 921, 9217, and 9270 showed no wall-climbing behavior. Homokaryons generally demonstrated weaker growth on the wheat grains, particularly the creeping-type strains, which exhibited extremely sparse mycelial development. However, homokaryons 9210, 9215, 9225, and 9228 displayed growth patterns comparable to those of heterokaryon 9270. The growth characteristics and morphological features exhibited by strains on wheat grains can differentiate some homokaryons from heterokaryons; however, they are insufficient to distinguish between the two types of homokaryons.

### Antagonistic reactions between SSIs and the heterokaryotic parent strain

The antagonistic reaction was a specific example of somatic incompatibility. Antagonism tests were conducted to confirm significant genetic differences between the SSIs and the parental strains. These tests involved 20 SSIs (including 17 homokaryotic strains and 3 heterokaryotic strains) and the heterokaryotic parent strain (Supplementary Fig. S4). Heterokaryotic strains 921 and the parent one exhibited distinct antagonistic lines, while strain 9217 showed harmonious growth with the heterokaryotic parent strain. Although strain 9270 did not produce antagonistic lines, it inhibited the colony growth. Homokaryotic strain 9210 demonstrated no visible antagonistic lines with the heterokaryotic parent strain and exhibited morphological similarity to strain 9270. Among the 17 antagonistic reactions between parent and homokaryotic strains, 14 pairs (excluding 9210, 9229, and 9231) displayed pronounced antagonism, with both sides of the Petri plates showing clear antagonistic phenomena. Of the 20 strains tested, 16 were successfully identified as homokaryons through antagonism, achieving an 80% success rate (Supplementary Fig. S4). This suggests the method's potential for preliminary homokaryon screening, although it cannot differentiate between the two types of homokaryons.

### Genomic heterozygosity ratios of homokaryotic and heterokaryotic strains

**Fig. 7:**
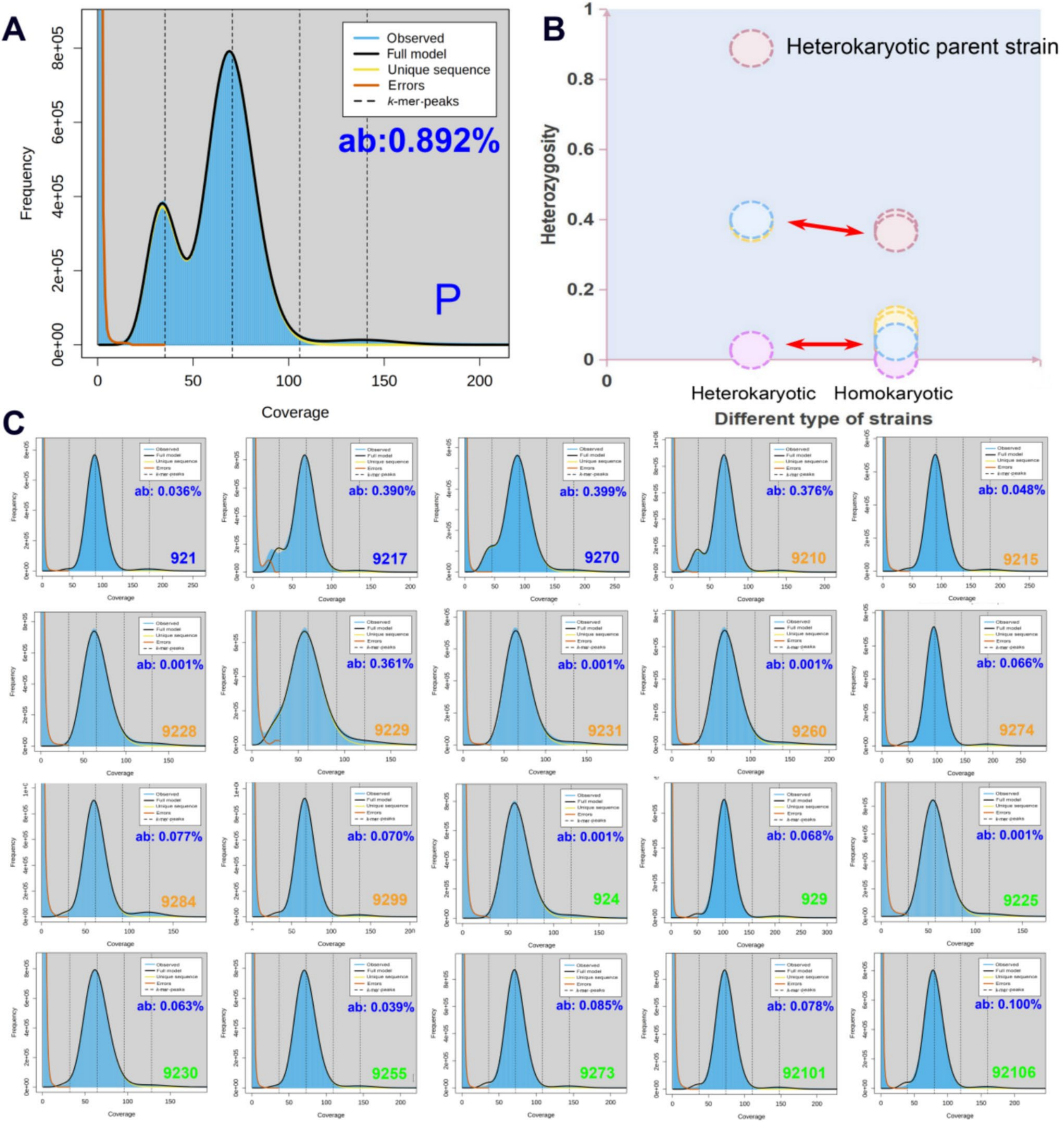
Heterozygosity ratio of heterokaryotic and homokaryotic strains. (**A**) Heterozygosity ratio of heterokaryotic parent strain. (**B**) Heterozygosity ratio of heterokaryotic and homokaryotic strains. (**C**) Heterozygosity ratio of SSIs strains. ab percentage of heterozygosity. P indicates the heterokaryotic parent strain. Blue labels indicate heterokaryotic strains; orange labels indicate homokaryotic strains with mating type *A1*; green labels indicate homokaryotic strains with mating type *A2*

All 21 strains were sequenced using the DNBSEQ-T7 platform to evaluate heterozygosity. *k*-mer analysis revealed distinct double peaks in heterokaryotic parent strains, whereas relatively low heterozygosity was observed in heterokaryotic strains isolated from basidiospores (Fig. 7). Additionally, all homokaryotic strains isolated from basidiospores exhibited low heterozygosity. However, some homokaryotic strains showed heterozygosity levels similar to those of heterokaryotic strains isolated from basidiospores, which was probably effected by genetic crossover, indicating that heterozygosity alone was not a highly accurate marker for distinguishing between homokaryotic and heterokaryotic strains. Notably, all strains with 0.001% heterozygosity were homokaryotic, suggesting that very low heterozygosity was characteristic of homokaryotic strains.

### Number of sterigmata and basidiospores observed by SEM

**Fig. 8:**
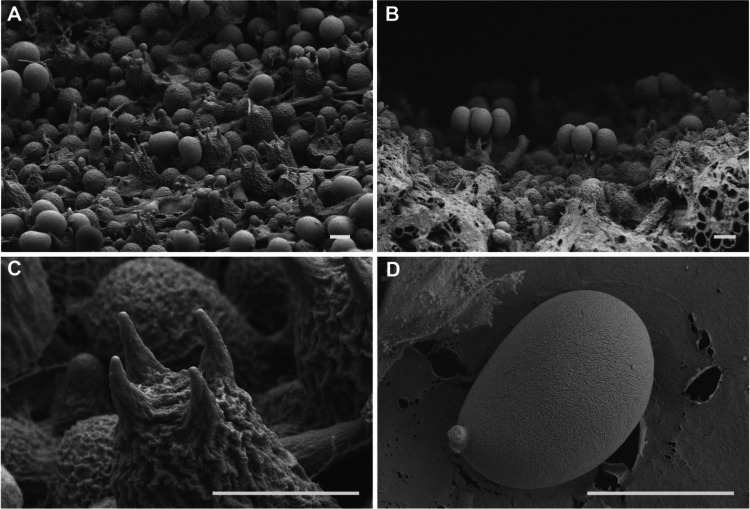
Hymenium morphology observed by SEM. (**A**) Hymenium structure. (**B**) Four-sterigmate basidia with four basidiospores. (**C**) Four-sterigmate basidium. (**D**) Basidiospores. Bars = 5 μm

To further understand the life cycle *of I. hispidus*, the hymenium was examined using SEM. SEM observations revealed that the hymenium contains only four-sterigma basidia, with each basidium producing four basidiospores (Fig. 8A, B and C). This finding aligns with the characteristics observed in most mushroom species.

### The inner structure of basidiospores observed by TEM

**Fig. 9:**
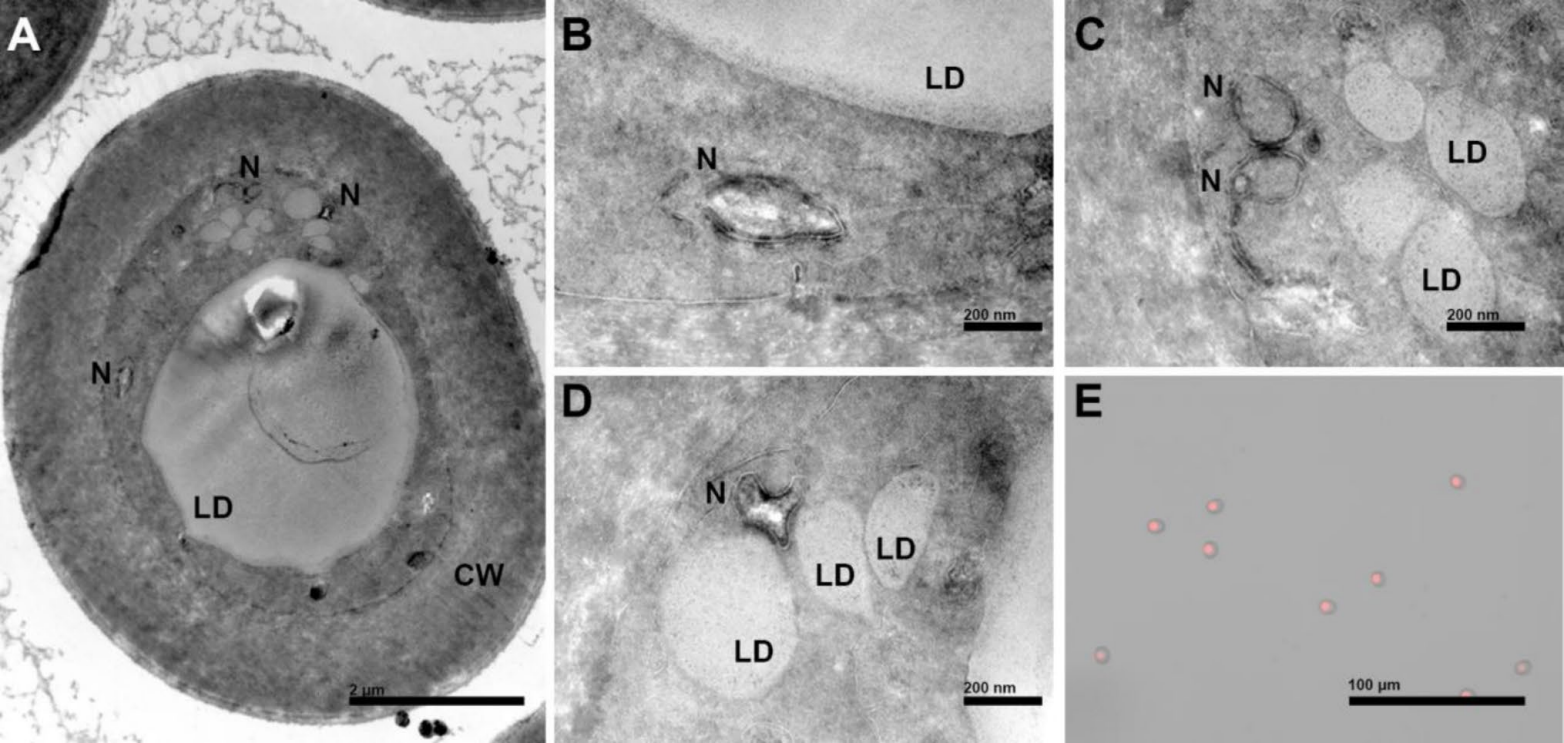
The inner structure of *I. hispidus* basidiospores observed by TEM. CW, cell wall; LD, lipid droplet; N, nucleus. (**A**) Entire basidiospore. (**B**, **C**, and **D**) Nucleus within the basidiospore. (**E**) Basidiospore were stained with Nile Red

Under TEM, the spore exhibited a relatively thick cell wall, measuring approximately 1–1.3 μm in thickness (Fig. 9A). The intracellular space was predominantly occupied by lipid droplets, as confirmed by Nile red staining, which revealed substantial lipid accumulation within the cytoplasm (Fig. 9 A, E). Nuclear structures were observed but displayed a dispersed distribution pattern; therefore, the number of nuclei in basidiospores remains ambiguous (Fig. 9B, C and D).

## Discussion

Wild Sanghuang requires specific natural ecological conditions for growth and basidiomata formation. The limited resources of Sanghuang have been overharvested, which falls far short of meeting market demand (Wu and Dai 2020; Yang et al. 2023). Therefore, the artificial cultivation of *I. hispidus* has become an essential approach to protect ecological resources and satisfy market needs. To support industrial development, breeding has become crucial. This study comprehensively elucidated the bipolar mating system of *I. hispidus* by integrating mating type loci analysis derived from genomic data with empirical mating tests. Furthermore, methods for identifying homokaryons were also characterized. These findings are expected to advance both genetic research and breeding practices associated with *I. hispidus*.

In previous studies, Kühner (1950) described the nuclear number in the mycelia of *I. hispidus* as coenocytic. However, recent research has described monokaryotic strains in the context of genomic sequencing, comparative genomic analysis, and metabolomic studies of *I. hispidus*. In the past few years, monokaryotic strains were reported to have germinated from basidiospores (Tang et al. 2022; Zhang et al. 2022; Wang et al. 2023; Bai et al. 2025; Ding et al. 2025). In this study, the nuclear number in the mycelia of homokaryotic and heterokaryotic strains was carefully examined using fluorescence staining of nuclei and septa. It was accurately determined that both homokaryotic and heterokaryotic strains were multinucleated. Therefore, the strains of *I. hispidus* should be described as multinuclear homokaryotic and multinuclear heterokaryotic. Additionally, although Song et al. (2022) reported observing clamp connections during microscopic examination of *I. hispidus* mycelia, we did not observe such structures in our study. Meanwhile, clamp connections were also not observed in other wild strains collected from Beijing City and Shaanxi Province, which is consistent with the earlier findings of Kühner (1950) and Stalpers (1978).

The mating system serves as a fundamental basis for conducting genetic studies and breeding programs. Consequently, the investigation of mating systems has gained prominence in the study of edible and medicinal mushrooms (Sonnenberg et al. 2017; Li et al. 2020; Li et al. 2025). Traditionally, the study of mating systems has involved a three-round mating test, which has been employed for the majority of species (Li et al. 2020; Zhang et al. 2023). However, advancements in genomics and molecular technologies have led to the development of more effective methodologies. Mating type loci have been identified as key regulators of mating; therefore, analysis of the mating type loci in compatible strains combine genomics and molecular biology can provide strong evidence regarding the mating system.

Consequently, these loci have become the primary focus of research, while mating tests have served as supplementary methods. However, some studies have opted to forgo mating tests altogether and infer the mating system based solely on one genomic information (Jiang et al. 2021; Zhang et al. 2022). For example, the observation that the potential *MAT-A* locus and the potential *MAT-B* locus were not located on the same contigs was used to suggest that the mating type of *I. hispidus* possesses a tetrapolar mating system (Zhang et al. 2022). This conclusion was unfounded and lacks sufficient scientific evidence. In the present study, both mating tests and analyses of mating type loci were conducted, confirming the bipolar mating system in *I. hispidus*.

In the traditional study of mating systems, in Basidiomycota, most were based on in vitro pairing experiments of cultures, where formation of intermingling mycelial zones, heterokaryons and clamp connections were used as diagnostic characters to test compatibility between homokaryons (Kauserud and Schumacher 2001). In view of the absence of clamp connections in *I. hispidus*, mating type genes were employed as molecular markers to identify whether mating had occurred. Four distinct mating phenomena were observed: aerial, gully, contact, and non-phenomenon. Among these, the aerial type was identified as the typical mating phenomenon. For instance, in fungi such as *A. bisporus*, *Agaricus sinodeliciosus*, and *W. hoelen*, the appearance of aerial hyphae was regarded as a signal of successful mating (Ling et al. 2019; Li et al. 2022). Moreover, the situation of successful mating with no observable phenomenon was also observed in *Sparassis latifolia* (Li et al. 2020). Notably, among the 21 verified mating combinations, the *A1* strain of 9215 and *A2* strain of 9225, which were from the aerial type, exhibited a contact type phenomenon but failed to mate successfully. The environment, such as the nutrients in the culture medium, is also a factor influencing mating (Mallett and Myrholm 1995).

Previously, due to limited understanding of genomic information and the high cost of sequencing, methods such as colony morphology, mycelial growth rate, auxotrophy, and isozyme analysis were used to distinguish homokaryotic and heterokaryotic strains in some mushrooms (Hansen 1979; Kerrigan et al. 1992). Subsequently, molecular markers such as simple sequence repeat (SSR), ITS, cleaved amplified polymorphic sequence (CAPS) and *rpb2* have been effectively utilized (Kauserud et al. 2001; Lim et al. 2008; Gao et al. 2013; Ling et al. 2019; Li et al. 2022). In this study, homokaryotic strains were identified by combining culture characteristics with molecular markers.

The growth rate and colony morphology were the most prominent morphological features for distinguishing homokaryotic and heterokaryotic strains. For example, in *A. bisporus*, homokaryotic strains exhibit clear differences in growth rate and colony morphology, a pattern also observed to *W. hoelen* (Li et al. 2021a). In *I. hispidus*, homokaryotic strains showed slower growth rates than heterokaryotic strains on PDA Petri plates. In previous studies, 6 weeks cultures were commonly used to observe cultural characteristics (Nobles 1948; Stalpers 1978). This experiment cultured homokaryotic and heterokaryotic strains only up to the 10th day. Additionally, growth performance on wheat grain substrate differed between homokaryotic and heterokaryotic strains. Antagonism reactions can also be used to differentiate homokaryotic strains; this method has been applied to *W. hoelen* and was useful for *I. hispidus*. However, antagonism tests and growth performance on wheat grain substrate may occasionally produce false positives. Molecular markers designed on mating type loci have also been used to distinguish homokaryotic strains. However, because mating type loci exhibit polymorphism across different strains or lineages, these molecular markers are not always reliable. Consequently, growth rate can serve as a highly accurate method for distinguishing homokaryotic strains in *I. hispidus*. It is important to note that this method may not always be effective, as some lineages of *W. hoelen* show no differences in growth rate or colony morphology between homokaryotic and heterokaryotic strains (Li et al. 2021a).

*k*-mer analysis, a fundamental method, has long been used to evaluate heterozygosity and genome size in genome sequencing (Kajitani et al. 2014). Initially, *k*-mer analysis was employed to distinguish between homokaryotic and heterokaryotic strains, providing a novel approach for further studies. The heterozygosity rate of heterokaryotic strains in *W. hoelen* was approximately 0.75%, whereas that of the homokaryotic strain was 0.01% (Li et al. 2021a). In this study, the heterozygosity ratios of the heterokaryotic parent strain, three heterokaryotic strains, and 17 homokaryotic strains were analyzed. The heterokaryotic parent strain exhibited high heterozygosity at 0.892%, while the three other heterokaryotic strains isolated from basidiospores showed heterozygosity values of 0.036%, 0.390%, and 0.399%, respectively. The heterozygosity of homokaryotic strains ranged from 0.001% to 0.376%. These results suggest that heterozygosity was not an effective marker for differentiating between homokaryotic and heterokaryotic strains, which contrasts with previous findings in *W. hoelen* (Li et al. 2021a). However, we also observed that very low heterozygosity (0.001%) may still serve as an indicator of homokaryotic strains.

This study reports for the first time that the basidiospores of *I. hispidus* exhibit autofluorescence, which has hindered the investigation of nuclear number in these basidiospores. To determine the nuclear distribution within the basidiospores, we attempted enzymatic digestion of the spore walls using lysozyme, cellulase, and other enzymes, followed by nuclear staining; however, these efforts were unsuccessful. According to previous records, the thick spore wall is a characteristic feature of *I. hispidus* spores (Cui et al. 2009). In this study, TEM revealed that the spore wall can be as thick as 1.3 μm. In species of the genus closely related to *I. hispidus*, basidiospores can be easily stained with 4′,6-diamidino-2-phenylindole (DAPI) as is the case of *Inonotus obliquus*, a species that does not exhibit thick spore walls (Sun et al. 2023). This suggests that the spore cell wall may be a significant factor affecting staining efficacy. Additionally, a large lipid droplet was observed inside the spores, and fluorescent staining confirmed this finding.

**Fig. 10:**
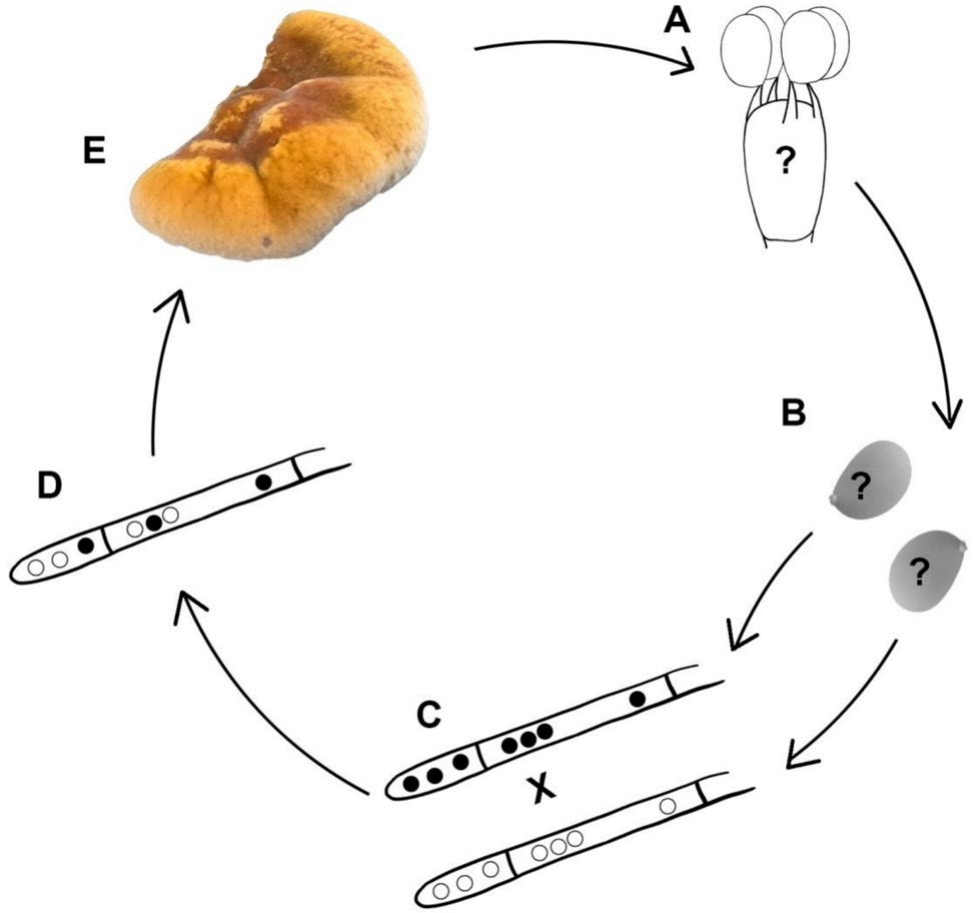
The life cycle of *I. hispidus*. (**A**) Basidia with four-basidiospores. (**B**) Basidiospores. (**C**) Mating of multinucleate homokaryons. (**D**) Multinucleate heterokaryotic hypha. (**E**) Fruiting body.? indicates the unknown nuclear number. X indicates mating

In the life cycles of *A. bisporus* and *W. hoelen*, which also possess bipolar mating systems, basidiospores can be mononucleate, binucleate or tetranucleate (Kamzolkina et al. 2006; Li et al. 2022). Although SEM confirmed that basidia produce four spores, and TEM observations revealed the internal spore structure, we speculate that the nuclei are not spherical and are located between the lipid droplet and the cell wall. However, due to the limited space for nuclear distribution and the irregular nuclear shapes, it was currently not possible to determine the exact number of nuclei per spore. The life cycle draft of *I. hispidus* is summarized in Fig. 10. However, the development of basidia, the nuclei formed within the basidia, and the number of cell nuclei in the spores remain unclear. Mature basidia produce four basidiospores (Fig. 10A). These basidiospores germinate and develop into multinucleated homokaryons (Fig. 10C). When homokaryotic SSIs mate with compatible ones, the heterokaryotic strain is formed and can fruit under suitable conditions (Fig. 10D, E). It is certain that the life cycle of *I. hispidus* involves heterothallism, but the complete life cycle of this fungus requires further elucidation.

## Conclusion

In conclusion, we first demonstrated the multinucleate characteristics of homokaryotic and heterokaryotic strains of *I. hispidus*, we revised the concept of monokaryotic and dikaryotic mycelia of *I. hispidus*. Furthermore, through genome sequencing and mating type analysis combined with traditional mating tests, we distinguished homokaryotic strains and revealed, for the first time, the bipolar of the mating system of *I. hispidus*, and rejected the previous tetrapolar result. Meanwhile, we carried out studies on phenotypes. A highly accurate method for distinguishing homokaryotic strains was established by using the growth rate of mycelia. In summary, this study revealed the bipolar mating system based on mating type loci and traditional mating tests, and developed reliable methods for identifying homokaryotic strains, which provided a new understanding of *I. hispidus*, and will promote superior strain breeding and industrial development of *I. hispidus*.

## Supplementary Information

Below is the link to the electronic supplementary material.

Supplementary File 1 (3.43 MB PDF)

## Data Availability

The resequencing datasets have been deposited in the NCBI Sequence Read Archive (SRA) under BioProject accessions PRJNA1314222. Additional data are available upon request.
