# Supplementary material for: Bipolar, not tetrapolar: mating system determination in Inonotus hispidus through genomic and phenotypic analysis
Source: Appl Microbiol Biotechnol. 2026 Jan 29;110:49. doi: 10.1007/s00253-026-13721-4 (PMC12858565; doi:10.1007/s00253-026-13721-4)
Supplement: Supplementary file 1 [file 253_2026_13721_MOESM1_ESM.pdf]

Supplemental Material

Bipolar, not tetrapolar: Mating system determination in *Inonotus hispidus* through genomic and phenotypic analysis

in Applied Microbiology and Biotechnology

Yanqi Chen[1], Shoujian Li[2], Jiao Zhang[1], Yuqing Jiang[1], Mengran Zhao[1], Zhihao Hou[1], Chenyang Huang[1]

[1] State Key Laboratory of Efficient Utilization of Arable Land in China, Institute of Agricultural Resources and Regional Planning, Chinese Academy of Agricultural Sciences, Beijing 100081, China

[2] Institute of Medicinal Plant Development, Chinese Academy of Medical Sciences and Peking Union Medical College, Beijing 100193, China

*Corresponding author: Chenyang Huang,

E-mail: huangchenyang@caas.cn

Telephone: +86-10-82106207

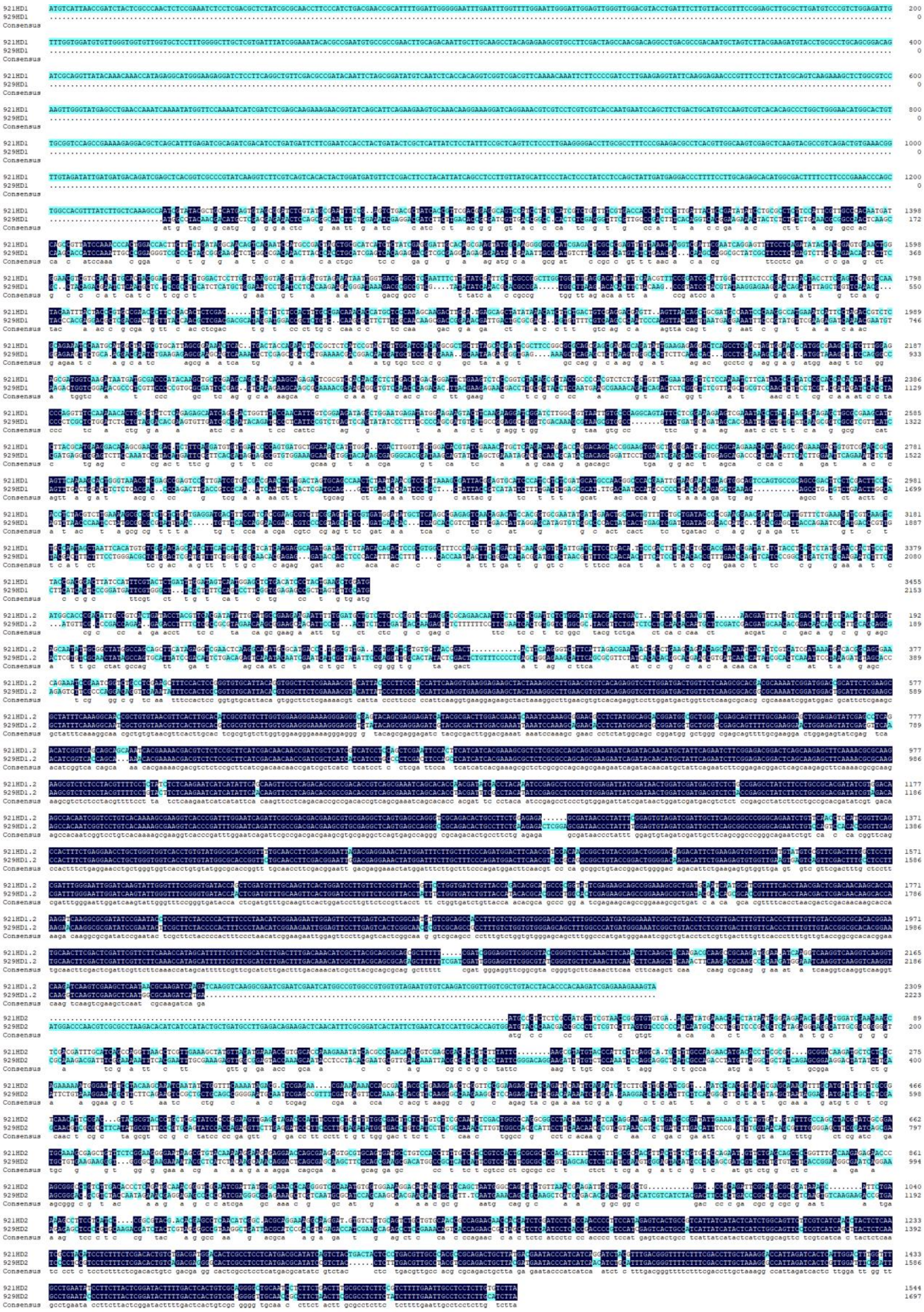

```
921pr1    ATGGCCCAAGATCCGACTTACCCGCTCTACCCAGTCGCCTTCTTCATCTCCCTCATCCTCGTCTTGATCCCTCTGCCGTGGCATCTTCAGGCCTGGAATGTTGGGACGTGCTCGTTCATGCTATGGACTGCTGCAGCTTGTCTCAGTTGGTTCGTCAATTCGATAGTTTGGAAGGACAACTTCATCAACCATGCGCCGGT   200
929pr1    ATGGCCCAAGATCCGACTTACCCGCTCTACCCAGTCGCCTTCTTCATCTCCCTCATCCTCGTCTTGATCCCTCTGCCGTGGCATCTTCAGGCCTGGAATGTTGGGACGTGCTCGTTCATGCTATGGACTGCTGCAGCTTGTCTCAGTTGGTTCGTCAATTCGATAGTTTGGAAGGACAACTTCATCAACCATGCGCCGGT   200
Consensusatggcccaagatccgacttacccgctctacccagtcgccttcttcatctccctcatcctcgtcttgatccctctgccgtggcatcttcaggcctggaatgttgggacgtgctcgttcatgctatggactgctgcagcttgtctcagttggttcgtcaattcgatagtttggaaggacaacttcatcaaccatgcgccggt

921pr1    TTGGTGCGATATTTCCTCGGCCCTCATAGTCGCGTACTCTGTGGCTCTTCCCGCGTCTTCCTTATGCATCACGCGACGACTCTTTAAGATTTCTCAAGTACAAACCGTCATGACCGGCCCGAAGGAGCGGCGAAAGGAACTTGCTATTGACTTGAGCCTCTGCGTGGGCTTGCCTGTGATTGTTCTGATCTTGCACTATT   400
929pr1    TTGGTGCGATATTTCCTCGGCCCTCATAGTCGCGTACTCTGTGGCTCTTCCCGCGTCTTCCTTATGCATCACGCGACGACTCTTTAAGATTTCTCAAGTACAAACCGTCATGACCGGCCCGAAGGAGCGGCGAAAGGAACTTGCTATTGACTTGAGCCTCTGCGTGGGCTTGCCTGTGATTGTTCTGATCTTGCACTATT   400
Consensusttggtgcgatatttcctcggccctcatagtcgcgtactctgtggctcttcccgcgtcttccttatgcatcacgcgacgactctttaagatttctcaagtacaaaccgtcatgaccggcccgaaggagcggcgaaaggaacttgctattgacttgagcctctgcgtgggcttgcctgtgattgttctgatcttgcactatt

921pr1    TCGTACAGGGACACCGGTTCGACATCTTCGAGCAAATCGGATGTTACCCTGTCACAGTCAACACGCCGCTGGCGTACCCGTTTGTGTGGATGTGGCCGTTACTTCTCGGGACGATCTCCATGATCTACTGCATCTTCACCGTCACATCCTTCCTGAAGCGCCGAAGAGAGATGAGCCAATTTCTCAGCAGCAATTCGCAA   600
929pr1    TCGTACAGGGACACCGGTTCGACATCTTCGAGCAAATCGGATGTTACCCTGTCACAGTCAACACGCCGCTGGCGTACCCGTTTGTGTGGATGTGGCCGTTACTTCTCGGGACGATCTCCATGATCTACTGCATCTTCACCGTCACATCCTTCCTGAAGCGCCGAAGAGAGATGAGCCAATTTCTCAGCAGCAATTCGCAA   600
Consensustcgtacagggacaccggttcgacatcttcgagcaaatcggatgttaccctgtcacagtcaacacgccgctggcgtacccgtttgtgtggatgtggccgttacttctcgggacgatctccatgatctactgcatcttcaccgtcacatccttcctgaagcgccgaagagagatgagccaatttctcagcagcaattcgcaa

921pr1    ATCACTTTCTCCCGATACTTCCGTCTTATGGCCTTGGCAACGATGGATACCCTGTTTACGGTCCCGCTTGCAACACTGGTGATAGTCCTGAACGCGACGGTTAACCCAATATATACTTGGAAGGGTCTTGATGACATCCACTGGGGTTTCAGTCGCGTTGAGACGGTCCCAGCGGTGTTTTGGCAGACCAACAGGTGGAC   800
929pr1    ATCACTTTCTCCCGATACTTCCGTCTTATGGCCTTGGCAACGATGGATACCCTGTTTACGGTCCCGCTTGCAACACTGGTGATAGTCCTGAACGCGACGGTTAACCCAATATATACTTGGAAGGGTCTTGATGACATCCACTGGGGTTTCAGTCGCGTTGAGACGGTCCCAGCGGTGTTTTGGCAGACCAACAGGTGGAC   800
Consensusatcactttctcccgatacttccgtcttatggccttggcaacgatggataccctgtttacggtcccgcttgcaacactggtgatagtcctgaacgcgacggttaacccaatatatacttggaagggtcttgatgacatccactggggtttcagtcgcgttgagacggtcccagcggtgttttggcagaccaacaggtggac

921pr1    CGTTGTCTCCTTCACGCTGAGCCGCGTATTCATCATCCTGTGTGCCGTTGTATTCTTCGTGTTCTTCGGGTTCGCAGAGGAGAATCGCAAGAACTACGCAAGGCTATACTGGAAGATCATGAAACGATTTGGTTACCAGCCAAAGCCCAAGGGGACGACGTTCACGAATTCGTCAGCTGTGCGGACACCGCATCTTCCAG   1000
929pr1    CGTTGTCTCCTTCACGCTGAGCCGCGTATTCATCATCCTGTGTGCCGTTGTATTCTTCGTGTTCTTCGGGTTCGCAGAGGAGAATCGCAAGAACTACGCAAGGCTATACTGGAAGATCATGAAACGATTTGGTTACCAGCCAAAGCCCAAGGGGACGACGTTCACGAATTCGTCAGCTGTGCGGACACCGCATCTTCCAG   1000
Consensuscgttgtctccttcacgctgagccgcgtattcatcatcctgtgtgccgttgtattcttcgtgttcttcgggttcgcagaggagaatcgcaagaactacgcaaggctatactggaagatcatgaaacgatttggttaccagccaaagcccaaggggacgacgttcacgaattcgtcagctgtgcggacaccgcatcttccag

921pr1    TCATGTCCGGTGGAGGCCAACATGGACTCAGGGTTCTTGTCAGCACAGATGCGAGAGCCGAGAAACGGGATTCGTTCATTTCATCGATTGGAGACCTTTCATCGTCGATATCTGTGAATTCGACGTATGACGAGCGAAAAGACTTTAGGTCGCCGATTGAGACGACGAGAACGGATCGGGACAGCCTACCACCGTCACCC   1200
929pr1    TCATGTCCGGTGGAGGCCAACATGGACTCAGGGTTCTTGTCAGCACAGATGCGAGAGCCGAGAAACGGGATTCGTTCATTTCATCGATTGGAGACCTTTCATCGTCGATATCTGTGAATTCGACGTATGACGAGCGAAAAGACTTTAGGTCGCCGATTGAGACGACGAGAACGGATCGGGACAGCCTACCACCGTCACCC   1200
Consensustcatgtccggtggaggccaacatggactcagggttcttgtcagcacagatgcgagagccgagaaacgggattcgttcatttcatcgattggagacctttcatcgtcgatatctgtgaattcgacgtatgacgagcgaaaagactttaggtcgccgattgagacgacgagaacggatcgggacagcctaccaccgtcaccc

921pr1    GATGATATAGAACTTCCGAACTTACATCATGTTCTTGCTACTACAACGACGACAACGACGGGGGCAGTAACATCGACACAACCGCCGGCTCGACCAGCGCGGCCGACCTCGCTAGGAGACGTTGTATA   1328
929pr1    GATGATATAGAACTTCCGAACTTACATCATGTTCTTGCTACTACAACGACGACAACGACGGGGGCAGTAACATCGACACAACCGCCGGCTCGACCAGCGCGGCCGACCTCGCTAGGAGACGTTGTATA   1328
Consensusgatgatatagaacttccgaacttacatcatgttcttgctactacaacgacgacaacgacgggggcagtaacatcgacacaaccgccggctcgaccagcgcggccgacctcgctaggagacgttgtata

921pr2    ATGTCTGCCTTCAGCATATTCTACTCGACACTCACCTTCTTTTCTTCCGCTCTCGCTTTATTTCTGCTCCCTTGGTTCGTTCGTTTTCGAAGTATACCTGGGCTGTTTGCGATTGGATGGCTTTTTGTGGTTAACCTCTTGTACGGGTTAAATGCGGCTGTTTTTACTGGTGATGGTAGCGGGGTCGGTGCTGGTGGGG   200
929pr2    ATGTCTGCCTTCAGCATATTCTACTCGACACTCACCTTCTTTTCTTCCGCTCTCGCTTTATTTCTGCTCCCTTGGTTCGTTCGTTTTCGAAGTATACCTGGGCTGTTTGCGATTGGATGGCTTTTTGTGGTTAACCTCTTGTACGGGTTAAATGCGGCTGTTTTTACTGGTGATGGTAGCGGGGTCGGTGCTGGTGGGG   200
Consensusatgtctgccttcagcatattctactcgacactcaccttcttttcttccgctctcgctttatttctgctcccttggttcgttcgttttcgaagtatacctgggctgtttgcgattggatggctttttgtggttaacctcttgtacgggttaaatgcggctgtttttactggtgatggtagcggggtcggtgctggtgggg

921pr2    GGCATCTAGGGCGGGCGATGAGAATATGCCGGTGCTGTTGAAGTTCCCTGCGTATTGCGATATTGTCACGAAGCTCCTTGTGGGGATGCAGATGGCCGTACCGACTGCCATGCTCTGCTATACCTTCTCCGTACATGATTGGATGGTCCGTCAACGGGAATGTGATACGAGCACGAAGCGCCTCGAATACAGAAGCCACT   400
929pr2    GGCATCTAGGGCGGGCGATGAGAATATGCCGGTGCTGTTGAAGTTCCCTGCGTATTGCGATATTGTCACGAAGCTCCTTGTGGGGATGCAGATGGCCGTACCGACTGCCATGCTCTGCTATACCTTCTCCGTACATGATTGGATGGTCCGTCAACGGGAATGTGATACGAGCACGAAGCGCCTCGAATACAGAAGCCACT   400
Consensusggcatctagggcgggcgatgagaatatgccggtgctgttgaagttccctgcgtattgcgatattgtcacgaagctccttgtggggatgcagatggccgtaccgactgccatgctctgctataccttctccgtacatgattggatggtccgtcaacgggaatgtgatacgagcacgaagcgcctcgaatacagaagccact

921pr2    TTCACGTCGCACTCTGTCTCCTTGCGCCGCTCGTATACCTGTCTCTTCACATCGCAGCGCAGGGGCATCGCTTCGATATAGTCGACAACGTCGGGTGTATTCCGTCGTCGTCGAGCACCGCATTGGGTTGGGTGCTCATTTGGCAGCTCCTTCTCGTTCCCTTCGCTGCTATACTCGTATACGGCTATTTCGTTTTCAGG   600
929pr2    TTCACGTCGCACTCTGTCTCCTTGCGCCGCTCGTATACCTGTCTCTTCACATCGCAGCGCAGGGGCATCGCTTCGATATAGTCGACAACGTCGGGTGTATTCCGTCGTCGTCGAGCACCGCATTGGGTTGGGTGCTCATTTGGCAGCTCCTTCTCGTTCCCTTCGCTGCTATACTCGTATACGGCTATTTCGTTTTCAGG   600
Consensusttcacgtcgcactctgtctccttgcgccgctcgtatacctgtctcttcacatcgcagcgcaggggcatcgcttcgatatagtcgacaacgtcgggtgtattccgtcgtcgtcgagcaccgcattgggttgggtgctcatttggcagctccttctcgttcccttcgctgctatactcgtatacggctatttcgttttcagg

921pr2    TATTCTTGGTCGCATTATCGCAGATACTGCGCGGAATACCTGACAACCTGGGAATTGGTCGACTCCGATCTGCTTTTCACGAACGCGCCGTTTGGGATCACACGCATTGTTCGGTTGTCTGCGATGATTGTCGCTGTAATGCTCTGTACTCTCGGTGCTCCGTATTACGCGCTCTCCGTGTTATCTCAAGCCGACGACAT   800
929pr2    TATTCTTGGTCGCATTATCGCAGATACTGCGCGGAATACCTGACAACCTGGGAATTGGTCGACTCCGATCTGCTTTTCACGAACGCGCCGTTTGGGATCACACGCATTGTTCGGTTGTCTGCGATGATTGTCGCTGTAATGCTCTGTACTCTCGGTGCTCCGTATTACGCGCTCTCCGTGTTATCTCAAGCCGACGACAT   800
Consensustattcttggtcgcattatcgcagatactgcgcggaatacctgacaacctgggaattggtcgactccgatctgcttttcacgaacgcgccgtttgggatcacacgcattgttcggttgtctgcgatgattgtcgctgtaatgctctgtactctcggtgctccgtattacgcgctctccgtgttatctcaagccgacgacat

921pr2    CGCGATTGCTAGAAGACCAGATTTCTATCGTGTCGAAGTCTTTAGCTCGCTGAACGATGCGTCTCCCAGCCTGACGAGCATGCGACTATTCCTGTGGTCAGGTCCGCTATATAGCATCCTCCTCTTCGCCGCTAGCGTGGATAAATCTGTCCTGCAATCATATCCCTTCATGATCAGGAAATATATCCTGCGACAGCGCG   1000
929pr2    CGCGATTGCTAGAAGACCAGATTTCTATCGTGTCGAAGTCTTTAGCTCGCTGAACGATGCGTCTCCCAGCCTGACGAGCATGCGACTATTCCTGTGGTCAGGTCCGCTATATAGCATCCTCCTCTTCGCCGCTAGCGTGGATAAATCTGTCCTGCAATCATATCCCTTCATGATCAGGAAATATATCCTGCGACAGCGCG   1000
Consensuscgcgattgctagaagaccagatttctatcgtgtcgaagtctttagctcgctgaacgatgcgtctcccagcctgacgagcatgcgactattcctgtggtcaggtccgctatatagcatcctcctcttcgccgctagcgtggataaatctgtcctgcaatcatatcccttcatgatcaggaaatatatcctgcgacagcgcg

921pr2    TCGGAGCGAACCCCGACCCGAGTCCAGGGATTGACCTCTCAACTCTCACACCAATCCCTGTACGGAGGCGAACTCACAGGCGTGTCATACGCAAGTCGCGCCAATACGACCCACCCCTGTCGGCGCTGGATTTCGATATCCCGCTTCCTCCAAGGCGTGATCCCGGATTCCTTAAGACTGCTTCACTTCCTCCTGTCCCG   1200
929pr2    TCGGAGCGAACCCCGACCCGAGTCCAGGGATTGACCTCTCAACTCTCACACCAATCCCTGTACGGAGGCGAACTCACAGGCGTGTCATACGCAAGTCGCGCCAATACGACCCACCCCTGTCGGCGCTGGATTTCGATATCCCGCTTCCTCCAAGGCGTGATCCCGGATTCCTTAAGACTGCTTCACTTCCTCCTGTCCCG   1200
Consensustcggagcgaaccccgacccgagtccagggattgacctctcaactctcacaccaatccctgtacggaggcgaactcacaggcgtgtcatacgcaagtcgcgccaatacgacccacccctgtcggcgctggatttcgatatcccgcttcctccaaggcgtgatcccggattccttaagactgcttcacttcctcctgtcccg

921pr2    AATACGCCTTTGGCCCCTGTCCGGGGCCCTCGAGAGCCGGAAATAGCGTACGTAAAGGACGTGGACGGTACCACTCGACGTATTCGGAATGCAAGAAGTGGCGAGTCTTTGGGTGCGATGCATTTACATGCACAGACGAACAGCAGATTTCCATTGTCCACTATTCTGGAAATACCGCCTATCCGTGAAAATAGCGGATG   1400
929pr2    AATACGCCTTTGGCCCCTGTCCGGGGCCCTCGAGAGCCGGAAATAGCGTACGTAAAGGACGTGGACGGTACCACTCGACGTATTCGGAATGCAAGAAGTGGCGAGTCTTTGGGTGCGATGCATTTACATGCACAGACGAACAGCAGATTTCCATTGTCCACTATTCTGGAAATACCGCCTATCCGTGAAAATAGCGGATG   1400
Consensusaatacgcctttggcccctgtccggggccctcgagagccggaaatagcgtacgtaaaggacgtggacggtaccactcgacgtattcggaatgcaagaagtggcgagtctttgggtgcgatgcatttacatgcacagacgaacagcagatttccattgtccactattctggaaataccgcctatccgtgaaaatagcggatg
```

```
921pr3   ATGATGGACGCGTATGACCCTCGCACGCATCCTCCCCCACACGTCCAATACGGCCAACACTCCTACGCTTCCTCGTACTACGCTCCTGACATCCACTCGTCGGCCTCCACCTCTTCATCTGCGTACCCGTCCCCTGCCTACCCTTCGTACGCCTACCAATCAGCATCTCCCTACAGTAGTGGTAACTCCGGGTACACCGC   200
929pr3   ATGATGGACGCGTATGACCCTCGCACGCATCCTCCCCCACACGTCCAATACGGCCAACACTCCTACGCTTCCTCGTACTACGCTCCTGACATCCACTCGTCGGCCTCCACCTCTTCATCTGCGTACCCGTCCCCTGCCTACCCTTCGTACGCCTACCAATCAGCATCTCCCTACAGTAGTGGTAACTCCGGGTACACCGC   200
Consensusatgatggacgcgtatgaccctcgcacgcatcctcccccacacgtccaatacggccaacactcctacgcttcctcgtactacgctcctgacatccactcgtcggcctccacctcttcatctgcgtacccgtcccctgcctacccttcgtacgcctaccaatcagcatctccctacagtagtggtaactccgggtacaccgc

921pr3   AGTTGAGTCGCAGACGTTGGGTTACTCCGTGCCATCCCAGAGTGCCGAGCCTTCCCTACCTAGTACTTTAACAGCGAGGGAAGGCGATCGCAGTATGGCAAGTAGCGCGCGCTCCTCCCCGGACGCAGGTCCTTCTCTTGACTATTCGTCAATGTCTTCCTCCGCTCCTAACCACCATCAGTCGCGTCCAATGGTGCACT   400
929pr3   AGTTGAGTCGCAGACGTTGGGTTACTCCGTGCCATCCCAGAGTGCCGAGCCTTCCCTACCTAGTACTTTAACAGCGAGGGAAGGCGATCGCAGTATGGCAAGTAGCGCGCGCTCCTCCCCGGACGCAGGTCCTTCTCTTGACTATTCGTCAATGTCTTCCTCCGCTCCTAACCACCATCAGTCGCGTCCAATGGTGCACT   400
Consensusagttgagtcgcagacgttgggttactccgtgccatcccagagtgccgagccttccctacctagtactttaacagcgagggaaggcgatcgcagtatggcaagtagcgcgcgctcctccccggacgcaggtccttctcttgactattcgtcaatgtcttcctccgctcctaaccaccatcagtcgcgtccaatggtgcact

921pr3   CTCAGTCGCACTCGCCCAATGCCTCAACATCCAGCAGCGCCAGTTCCTCACCTCGTCCCTCACAGCAGCCTCAGATCAGTTCTCTCCCCATAGCCCCCTCCGCCCTCCCTCATAACTCGGCTGGGTCCCATAACCAGATCACGTTCAATGGGACACCGCTCTCACGGCCTCTGACTCACAAGGAGCAGGAGATGCTCGCA   600
929pr3   CTCAGTCGCACTCGCCCAATGCCTCAACATCCAGCAGCGCCAGTTCCTCACCTCGTCCCTCACAGCAGCCTCAGATCAGTTCTCTCCCCATAGCCCCCTCCGCCCTCCCTCATAACTCGGCTGGGTCCCATAACCAGATCACGTTCAATGGGACACCGCTCTCACGGCCTCTGACTCACAAGGAGCAGGAGATGCTCGCA   600
Consensusctcagtcgcactcgcccaatgcctcaacatccagcagcgccagttcctcacctcgtccctcacagcagcctcagatcagttctctccccatagccccctccgccctccctcataactcggctgggtcccataaccagatcacgttcaatgggacaccgctctcacggcctctgactcacaaggagcaggagatgctcgca

921pr3   CATCTTGATCGCCTCAAGTTCTTCCTCGCGACTGCTCCGAGCCGCTGGGCAACAGCGGACGGCGACGGGCAAGGAACACCGATGATGCCTCACCCAAATACTCACCCCGCACTGAACCGCTTCTTGTTGCCGTCTGGTGAATACGTGACCTGTGTGCTCTGGTCAGGGCTGTACCATATCACTGGAACTGACATTGTCCG   800
929pr3   CATCTTGATCGCCTCAAGTTCTTCCTCGCGACTGCTCCGAGCCGCTGGGCAACAGCGGACGGCGACGGGCAAGGAACACCGATGATGCCTCACCCAAATACTCACCCCGCACTGAACCGCTTCTTGTTGCCGTCTGGTGAATACGTGACCTGTGTGCTCTGGTCAGGGCTGTACCATATCACTGGAACTGACATTGTCCG   800
Consensuscatcttgatcgcctcaagttcttcctcgcgactgctccgagccgctgggcaacagcggacggcgacgggcaaggaacaccgatgatgcctcacccaaatactcaccccgcactgaaccgcttcttgttgccgtctggtgaatacgtgacctgtgtgctctggtcagggctgtaccatatcactggaactgacattgtccg

921pr3   CGCTCTCGTATTTCGATTCGAGGCTTTCGGTCGTCCTGTACGCAACATGAAGAAGTTCGAGGAAGGCGTATTCTCCGACCTCCGTAACTTGAAGCCTGGGACAGACGCCTGCCTTGAAGAGCCAAAGTCCCCATTCCTCGATCTTCTGTTCAAGTATCAATGCATCAGGACGCAGAAGAAGCAGAAGGTCTTCTATTGGC   1000
929pr3   CGCTCTCGTATTTCGATTCGAGGCTTTCGGTCGTCCTGTACGCAACATGAAGAAGTTCGAGGAAGGCGTATTCTCCGACCTCCGTAACTTGAAGCCTGGGACAGACGCCTGCCTTGAAGAGCCAAAGTCCCCATTCCTCGATCTTCTGTTCAAGTATCAATGCATCAGGACGCAGAAGAAGCAGAAGGTCTTCTATTGGC   1000
Consensuscgctctcgtatttcgattcgaggctttcggtcgtcctgtacgcaacatgaagaagttcgaggaaggcgtattctccgacctccgtaacttgaagcctgggacagacgcctgccttgaagagccaaagtccccattcctcgatcttctgttcaagtatcaatgcatcaggacgcagaagaagcagaaggtcttctattggc

921pr3   TGTTCCTGGATGCCCTTGAGCGTGATTTGAAGCGCGAGAAGATGGGCCTGGAACCGACAACGGTTGTAGTCGGCGAACCTGCTCTGTCGTTTAGGTACGACAGTAAGCGCTCTCTATACGAGCAGTTCTCGAAAGCGCAAGGCATTCAAGATGGCGAAGGTGAACTGGAATCCGCTGTTCGTCGTGCGGAAGAGGCAATG   1200
929pr3   TGTTCCTGGATGCCCTTGAGCGTGATTTGAAGCGCGAGAAGATGGGCCTGGAACCGACAACGGTTGTAGTCGGCGAACCTGCTCTGTCGTTTAGGTACGACAGTAAGCGCTCTCTATACGAGCAGTTCTCGAAAGCGCAAGGCATTCAAGATGGCGAAGGTGAACTGGAATCCGCTGTTCGTCGTGCGGAAGAGGCAATG   1200
Consensustgttcctggatgcccttgagcgtgatttgaagcgcgagaagatgggcctggaaccgacaacggttgtagtcggcgaacctgctctgtcgtttaggtacgacagtaagcgctctctatacgagcagttctcgaaagcgcaaggcattcaagatggcgaaggtgaactggaatccgctgttcgtcgtgcggaagaggcaatg

921pr3   GCTATGGAGCAAGAGAGCGAAGCTCGAAGAGTGGCCTCAGGATCTACTGGGAGCTCTGAGCGTGATGTTGCACCCAGCACTATCTTCAACATGTTCTCACTGTTTGAGGGTAGCCCAAACTACAAGCAACGCCGCAAGAAGGGTCCGCGCACGGGACGCAGTGGCCTTGGACGTGATTCTGCATCGGAGGAAGAGCGCGA   1400
929pr3   GCTATGGAGCAAGAGAGCGAAGCTCGAAGAGTGGCCTCAGGATCTACTGGGAGCTCTGAGCGTGATGTTGCACCCAGCACTATCTTCAACATGTTCTCACTGTTTGAGGGTAGCCCAAACTACAAGCAACGCCGCAAGAAGGGTCCGCGCACGGGACGCAGTGGCCTTGGACGTGATTCTGCATCGGAGGAAGAGCGCGA   1400
Consensusgctatggagcaagagagcgaagctcgaagagtggcctcaggatctactgggagctctgagcgtgatgttgcacccagcactatcttcaacatgttctcactgtttgagggtagcccaaactacaagcaacgccgcaagaagggtccgcgcacgggacgcagtggccttggacgtgattctgcatcggaggaagagcgcga

921pr3   AATGCACGGCGGTGCTGGATCAGGGGATGTCGTAGAAGATGTCAACGGCGAGATGAGCGCAGCTGCAATGTTTGACGCTCAGGCCGGTCTCGGTGGCCATCAAGCGAGAGCACAACGTCAAGCCGAGAGTCAGCGTGCTATCCAAGCGCAGCGTCTCGCAATCATCAACCAGCAAACGAATTCTATCTCCAAGACGCGAC   1600
929pr3   AATGCACGGCGGTGCTGGATCAGGGGATGTCGTAGAAGATGTCAACGGCGAGATGAGCGCAGCTGCAATGTTTGACGCTCAGGCCGGTCTCGGTGGCCATCAAGCGAGAGCACAACGTCAAGCCGAGAGTCAGCGTGCTATCCAAGCGCAGCGTCTCGCAATCATCAACCAGCAAACGAATTCTATCTCCAAGACGCGAC   1600
Consensusaatgcacggcggtgctggatcaggggatgtcgtagaagatgtcaacggcgagatgagcgcagctgcaatgtttgacgctcaggccggtctcggtggccatcaagcgagagcacaacgtcaagccgagagtcagcgtgctatccaagcgcagcgtctcgcaatcatcaaccagcaaacgaattctatctccaagacgcgac

921pr3   AATTCCCTGGTCGTGCAATGCACCCATCGTTAAGCAGTTCACAATTGCACAGCAACTCGCTTCCAGCGAACTTTGACACAGGACGCAAGACGAGGGCGTATGTTTGTCCGCTGTACTCATGCCAGCGCCTGTTCAAGCGCCTCGAGCATTTGAAGCGGCACGTTCGGATGCACACGATGGAGCGGCCACACCAGTGCGAA   1800
929pr3   AATTCCCTGGTCGTGCAATGCACCCATCGTTAAGCAGTTCACAATTGCACAGCAACTCGCTTCCAGCGAACTTTGACACAGGACGCAAGACGAGGGCGTATGTTTGTCCGCTGTACTCATGCCAGCGCCTGTTCAAGCGCCTCGAGCATTTGAAGCGGCACGTTCGGATGCACACGATGGAGCGGCCACACCAGTGCGAA   1800
Consensusaattccctggtcgtgcaatgcacccatcgttaagcagttcacaattgcacagcaactcgcttccagcgaactttgacacaggacgcaagacgagggcgtatgtttgtccgctgtactcatgccagcgcctgttcaagcgcctcgagcatttgaagcggcacgttcggatgcacacgatggagcggccacaccagtgcgaa

921pr3   GTGTGCCAGCGCCGATTCTCGCGAGCTGATAATCTCAACCAGCACATGCGCACCCACTCGCGCGGTGGTAACGTCAACATGTTGCCAGTTGGTGATATAGACGGGGATGAGGGTGTTGATCAAGTGATGTACGCACCTGGCGAGCAGTACGACCTGAATGCATGCGAAATTGAGGCGGATGCATCGATGCGTTTTGAGGA   2000
929pr3   GTGTGCCAGCGCCGATTCTCGCGAGCTGATAATCTCAACCAGCACATGCGCACCCACTCGCGCGGTGGTAACGTCAACATGTTGCCAGTTGGTGATATAGACGGGGATGAGGGTGTTGATCAAGTGATGTACGCACCTGGCGAGCAGTACGACCTGAATGCATGCGAAATTGAGGCGGATGCATCGATGCGTTTTGAGGA   2000
Consensusgtgtgccagcgccgattctcgcgagctgataatctcaaccagcacatgcgcacccactcgcgcggtggtaacgtcaacatgttgccagttggtgatatagacggggatgagggtgttgatcaagtgatgtacgcacctggcgagcagtacgacctgaatgcatgcgaaattgaggcggatgcatcgatgcgttttgagga

921pr3   CGACGAGTATACAGGCTCTTATGTTGCGCCCTCATCGGCACCCAACGGCTCTCCCTACAGTAGTTTCGTTAGCGCAGAGAACTCTCAGTTCACTAATTTGGTGACGAGTCCTGAAAATTCGCCTCGGTTGACTGAGAGCAACTGGTCGGCGACACACGGTCATTCAAACTCGTTGCCAGCTGGCTACGACTACGGATCTT   2200
929pr3   CGACGAGTATACAGGCTCTTATGTTGCGCCCTCATCGGCACCCAACGGCTCTCCCTACAGTAGTTTCGTTAGCGCAGAGAACTCTCAGTTCACTAATTTGGTGACGAGTCCTGAAAATTCGCCTCGGTTGACTGAGAGCAACTGGTCGGCGACACACGGTCATTCAAACTCGTTGCCAGCTGGCTACGACTACGGATCTT   2200
Consensuscgacgagtatacaggctcttatgttgcgccctcatcggcacccaacggctctccctacagtagtttcgttagcgcagagaactctcagttcactaatttggtgacgagtcctgaaaattcgcctcggttgactgagagcaactggtcggcgacacacggtcattcaaactcgttgccagctggctacgactacggatctt

921pr3   CGCATCCGTCACCGGCGTTCAGTACCTCCAGCGCGCCGTCTTCACGGTTGGCGACTGCGTACAGCAATCCAACGTACTCTGTCCCACACGAGGTCCCCTCCAACTCCTCATCGTATGCCGCTTCTTCGGCCGGCTCGCTTTCGGCGCCTGCGCATAAGCAATCGTTCGACCACGCCGCACTGTTGGCCCCATCACTTGGA   2400
929pr3   CGCATCCGTCACCGGCGTTCAGTACCTCCAGCGCGCCGTCTTCACGGTTGGCGACTGCGTACAGCAATCCAACGTACTCTGTCCCACACGAGGTCCCCTCCAACTCCTCATCGTATGCCGCTTCTTCGGCCGGCTCGCTTTCGGCGCCTGCGCATAAGCAATCGTTCGACCACGCCGCACTGTTGGCCCCATCACTTGGA   2400
Consensuscgcatccgtcaccggcgttcagtacctccagcgcgccgtcttcacggttggcgactgcgtacagcaatccaacgtactctgtcccacacgaggtcccctccaactcctcatcgtatgccgcttcttcggccggctcgctttcggcgcctgcgcataagcaatcgttcgaccacgccgcactgttggccccatcacttgga

921pr3   AGTGTGCAGGGCTTCTCTACAAGTGCCGTCGGTCCGGTGCGCCGTTACCGCAGCGTCACCCCGACCATTGCACGTACCGGGGAGCAGATCCGTCGTCCACTAACTGCGAATACGCTTGACGCAAGTCCGCTCGGACCGGGTGCCGTGACTTCGCGCGGTTACCATCCCTATTCTTACGCTTCTGTTTCTCAGCACTCGAG   2600
929pr3   AGTGTGCAGGGCTTCTCTACAAGTGCCGTCGGTCCGGTGCGCCGTTACCGCAGCGTCACCCCGACCATTGCACGTACCGGGGAGCAGATCCGTCGTCCACTAACTGCGAATACGCTTGACGCAAGTCCGCTCGGACCGGGTGCCGTGACTTCGCGCGGTTACCATCCCTATTCTTACGCTTCTGTTTCTCAGCACTCGAG   2600
Consensusagtgtgcagggcttctctacaagtgccgtcggtccggtgcgccgttaccgcagcgtcaccccgaccattgcacgtaccggggagcagatccgtcgtccactaactgcgaatacgcttgacgcaagtccgctcggaccgggtgccgtgacttcgcgcggttaccatccctattcttacgcttctgtttctcagcactcgag

921pr3   CCCAGCAGCGTACCAGACTCAACTTGACTATTCGCAGCACACACCCGGTACGCAGTCGAATTCACCCGCAGGCTCAACAGTCTACCAAGAGGACTTTCAAACGTTGGTTGGGCTAGATGCAGCGACTCTTGGCGGTTACGAGAGTAACTCGATTGCGGCATCGGGTGTTGCGGCGTACGCGAGTGATGGATATTCGGAAC   2800
929pr3   CCCAGCAGCGTACCAGACTCAACTTGACTATTCGCAGCACACACCCGGTACGCAGTCGAATTCACCCGCAGGCTCAACAGTCTACCAAGAGGACTTTCAAACGTTGGTTGGGCTAGATGCAGCGACTCTTGGCGGTTACGAGAGTAACTCGATTGCGGCATCGGGTGTTGCGGCGTACGCGAGTGATGGATATTCGGAAC   2800
Consensuscccagcagcgtaccagactcaacttgactattcgcagcacacacccggtacgcagtcgaattcacccgcaggctcaacagtctaccaagaggactttcaaacgttggttgggctagatgcagcgactcttggcggttacgagagtaactcgattgcggcatcgggtgttgcggcgtacgcgagtgatggatattcggaac

921pr3   CAGCGCCGCAGGGACAATTCACTGCCTCGGCGGATTCCGCAGCAGCGGCGACTAATTACTATGGGGATCTGGGCAATCTGAACGGGACCGCGCAATTCTCGGAACAGTCATACTCCTATGCGTCCTA   2927
929pr3   CAGCGCCGCAGGGACAATTCACTGCCTCGGCGGATTCCGCAGCAGCGGCGACTAATTACTATGGGGATCTGGGCAATCTGAACGGGACCGCGCAATTCTCGGAACAGTCATACTCCTATGCGTCCTA   2927
Consensuscagcgccgcagggacaattcactgcctcggcggattccgcagcagcggcgactaattactatggggatctgggcaatctgaacgggaccgcgcaattctcggaacagtcatactcctatgcgtccta
```

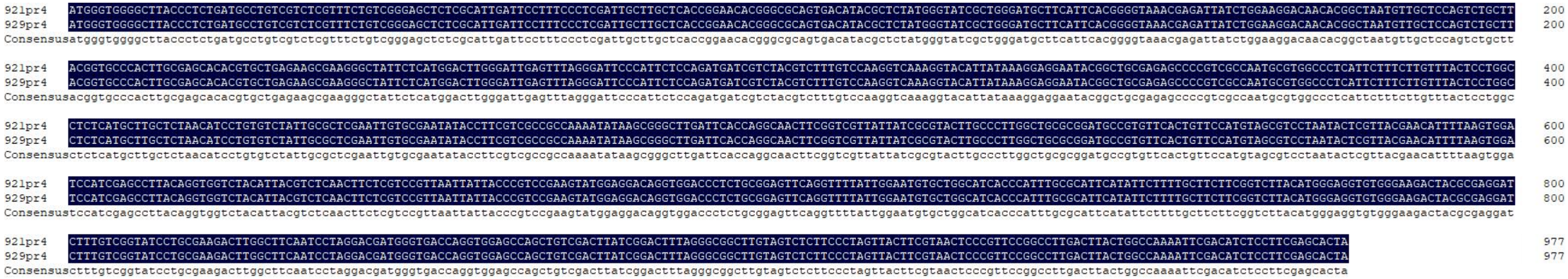

Fig. S1 Nucleotide sequence alignment of three *HD* and four *PR* genes between strains 921 and 929. The sequences labeled 921 in the sequence name were from strain 921, while labeled 929 in the sequence name were from strain 929.

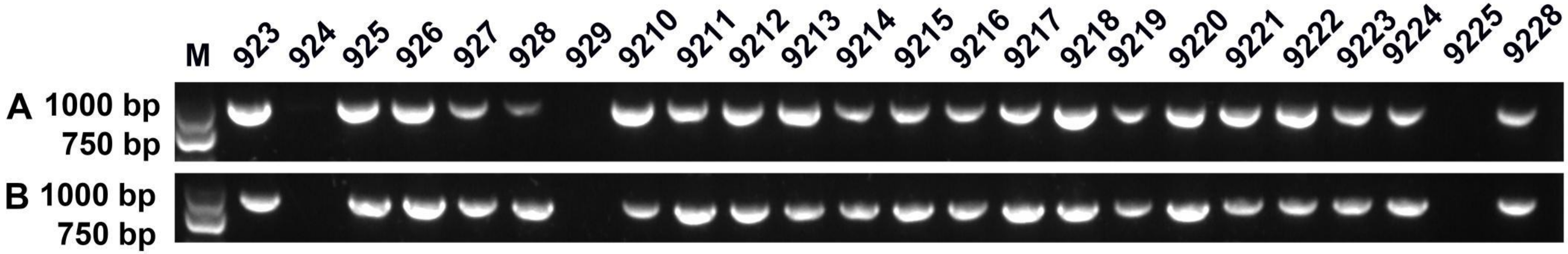

Fig. S2 PCR verification of the linkage of mating type genes *HD1* and *HD1.2*. **(A)** Strains that successfully amplified with primers A1-HD1F1/R1. **(B)** Strains that successfully amplified with primers A1-HD1.2F1/R1. M indicates the 2-kb size marker.

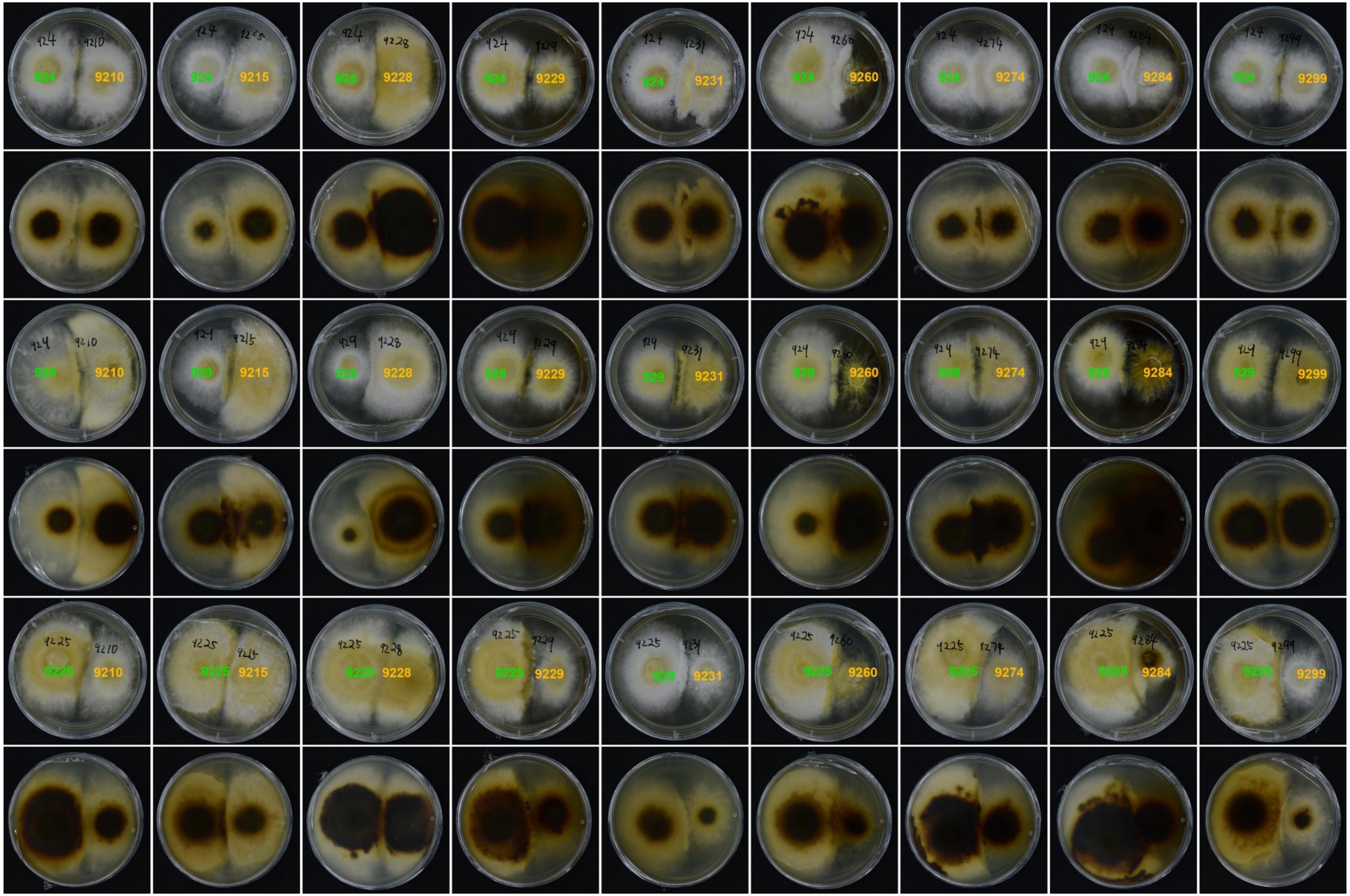

924 9210
924 9215
924 9228
924 9229
924 9231
924 9260
924 9274
924 9284
924 9299
929 9210
929 9215
929 9228
929 9229
929 9231
929 9260
929 9274
929 9284
929 9299
9225 9210
9225 9215
9225 9228
9225 9229
929 9231
9225 9260
9225 9274
9225 9284
9225 9299

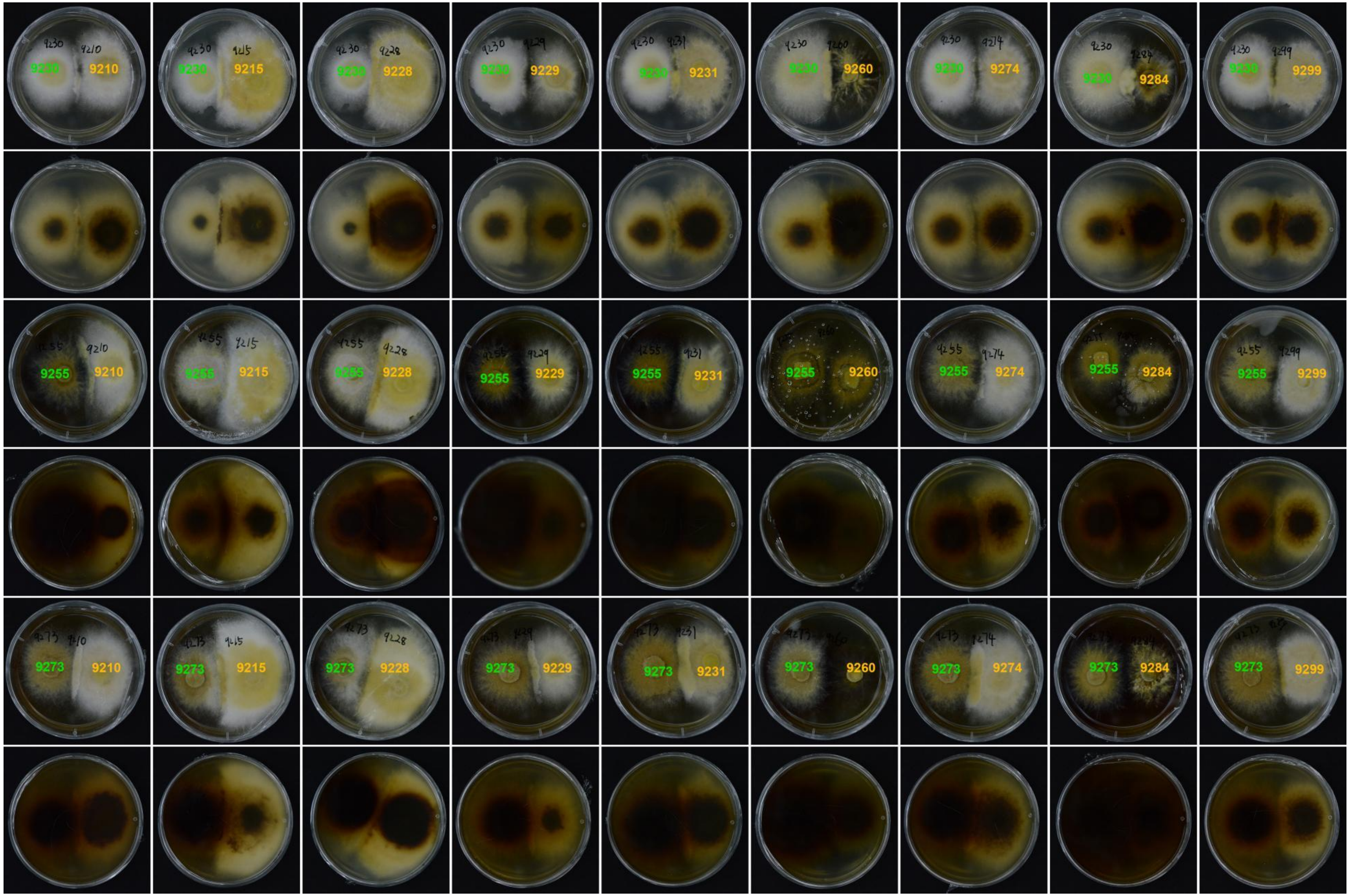
9230 9210
9230 9215
9230 9228
9230 9229
9230 9231
9230 9260
9230 9274
9230 9284
9230 9299
9255 9210
9255 9215
9255 9228
9255 9229
9255 9231
9255 9260
9255 9274
9255 9284
9255 9299
9273 9210
9273 9215
9273 9228
9273 9229
9273 9231
9273 9260
9273 9274
9273 9284
9273 9299

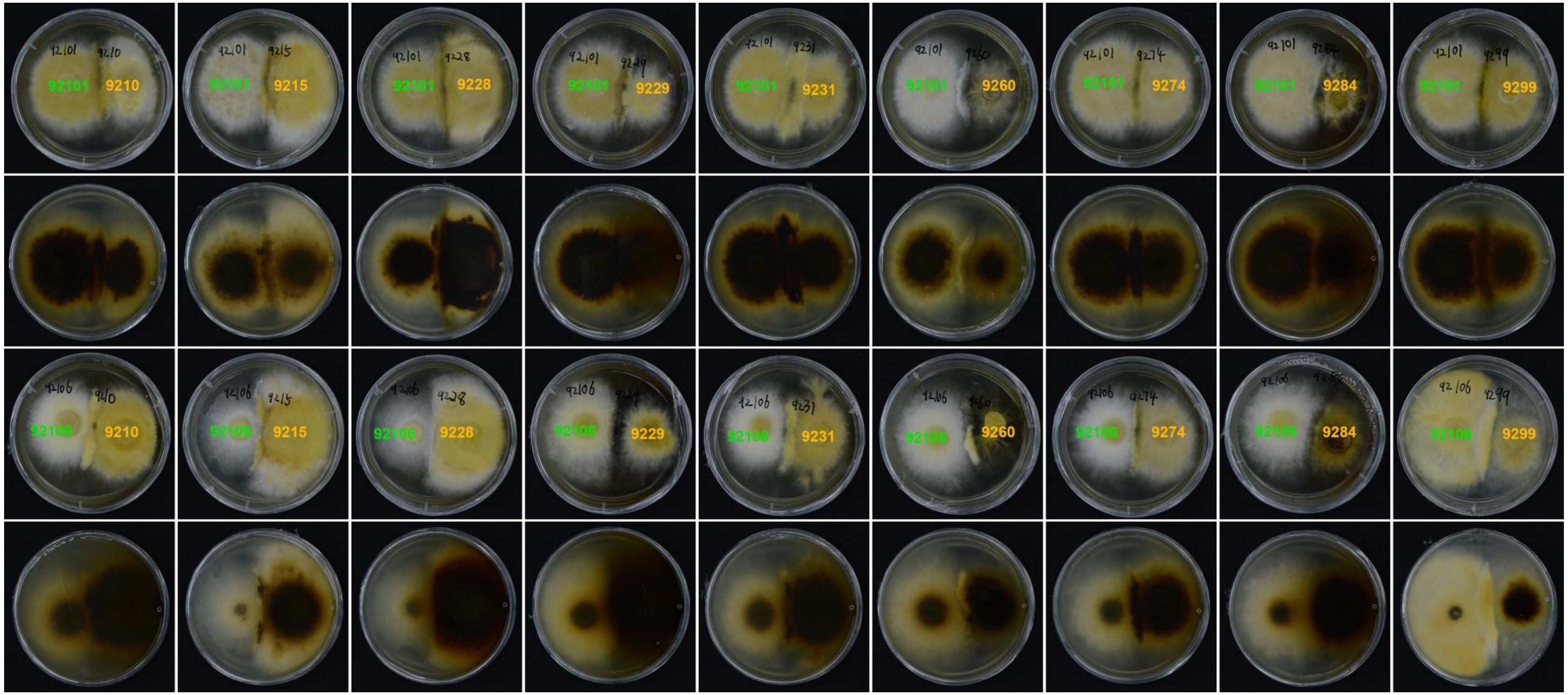

Fig. S3 Mating reactions of SSIs. Qrange labels indicate homokaryotic strains with mating type *A1*; green labels indicate homokaryotic strains with mating type *A2*.

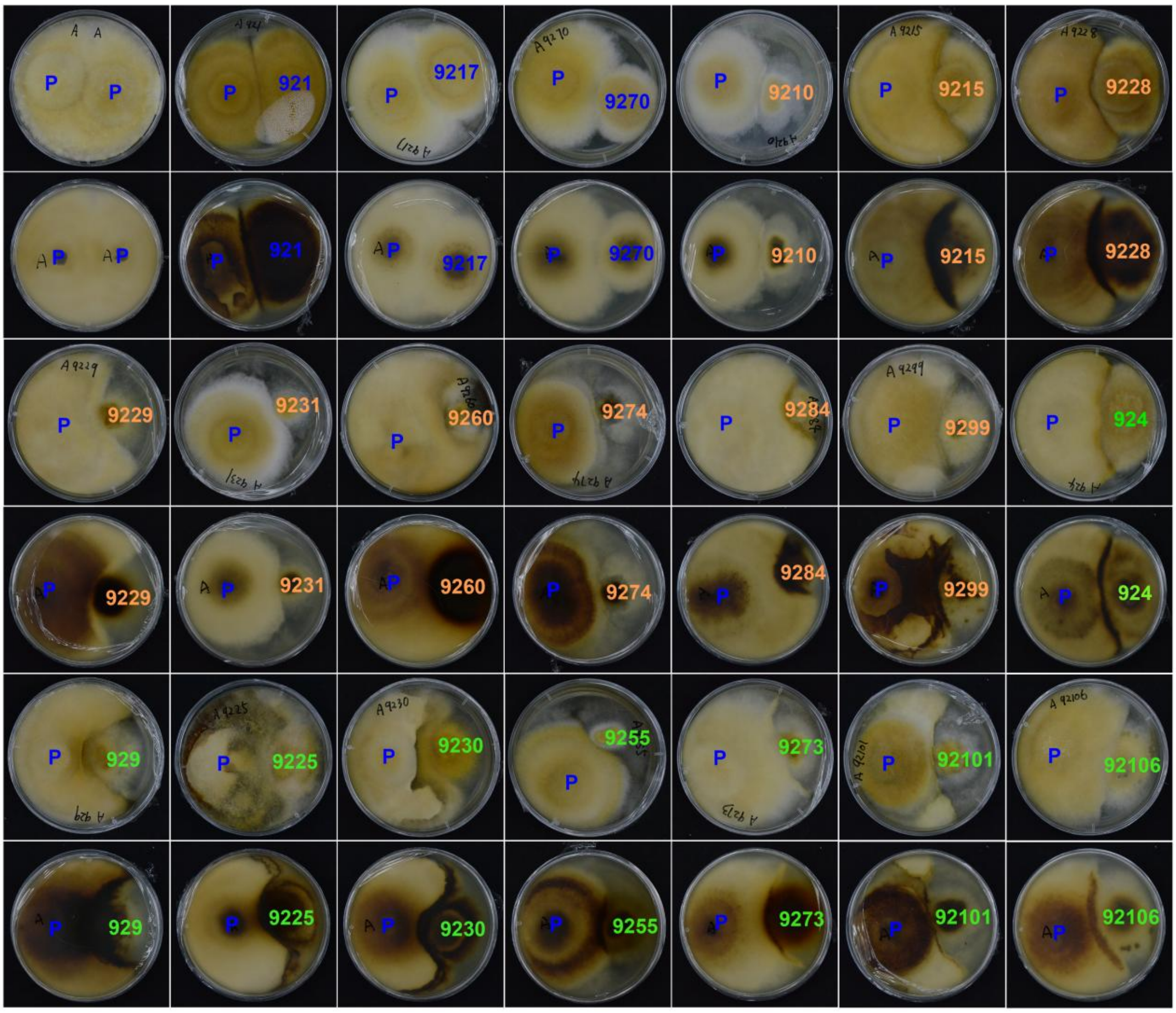

Fig. S4 Antagonistic reactions between the heterokaryotic parent strain and single spore strains. P indicates the heterokaryotic parent strain. Blue labels indicate heterokaryotic strains; orange labels indicate homokaryotic strains with mating type *A1*; green labels indicate homokaryotic strains with mating type *A2*.
