# Supplementary material for: Bipolar, not tetrapolar: mating system determination in Inonotus hispidus through genomic and phenotypic analysis
Source: Appl Microbiol Biotechnol. 2026 Jan 29;110(1):49. doi: 10.1007/s00253-026-13721-4 (PMC12858565; doi:10.1007/s00253-026-13721-4)
Supplement: Supplementary file 1 — (3.43 MB PDF) [file 253_2026_13721_MOESM1_ESM.pdf]

Supplemental Material

Bipolar, not tetrapolar: Mating system determination in *Inonotus hispidus* through genomic and phenotypic analysis

in Applied Microbiology and Biotechnology

Yanqi Chen[1], Shoujian Li[2], Jiao Zhang[1], Yuqing Jiang[1], Mengran Zhao[1], Zhihao Hou[1], Chenyang Huang[1]

[1] State Key Laboratory of Efficient Utilization of Arable Land in China, Institute of Agricultural Resources and Regional Planning, Chinese Academy of Agricultural Sciences, Beijing 100081, China

[2] Institute of Medicinal Plant Development, Chinese Academy of Medical Sciences and Peking Union Medical College, Beijing 100193, China

*Corresponding author: Chenyang Huang,

E-mail: huangchenyang@caas.cn

Telephone: +86-10-82106207

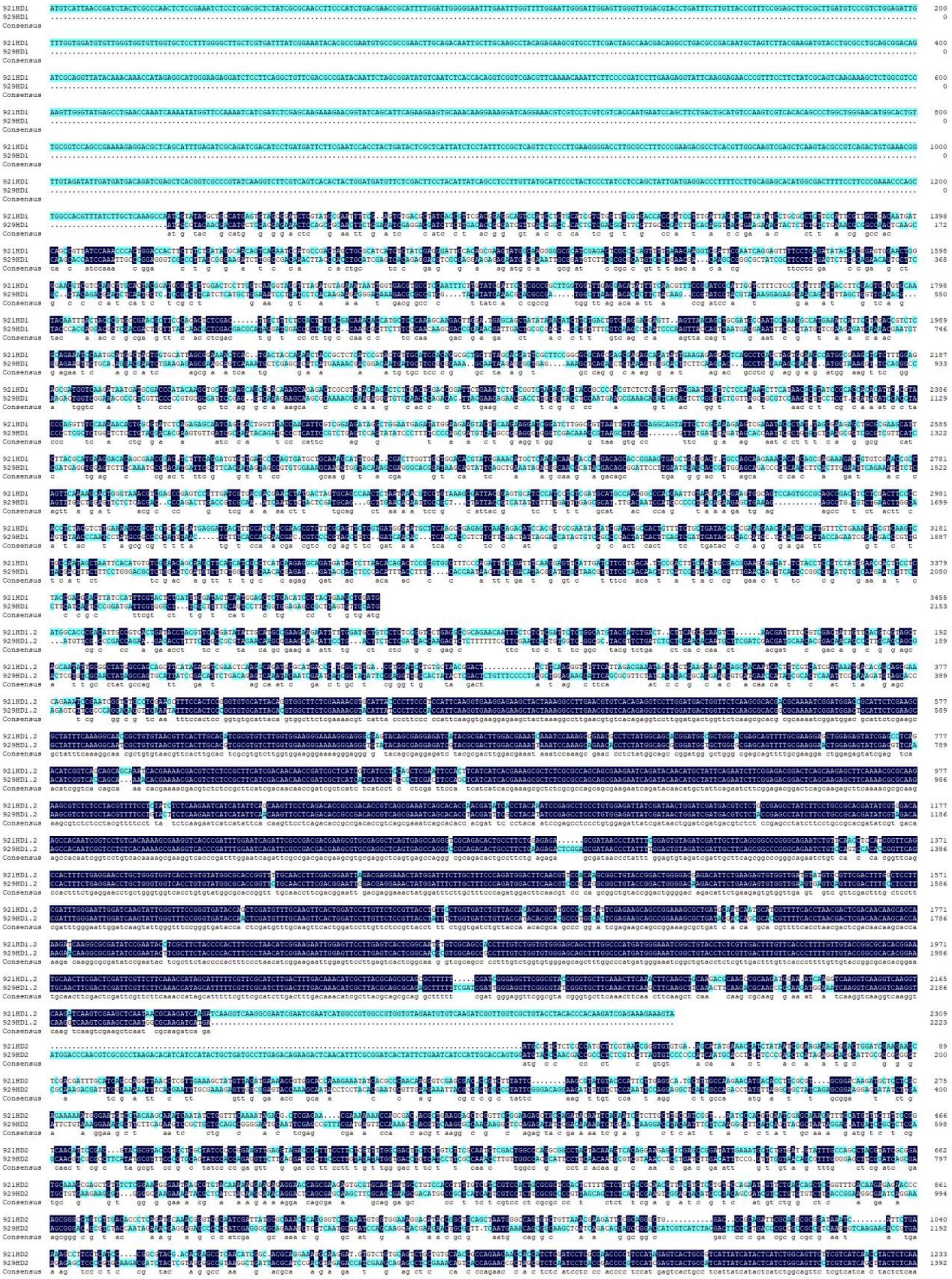
921HD1
929HD1
921HD1.2
929HD1.2
921HD2
929HD2
Consensus

```
921pr1   ATGGCCCAAGATCCGACTTACCCGCTCTACCCAGTCGCCTTCTTCATCTCCCTCATCCTCGTCTTGATCCCTCTGCCGTGGCATCTTCAGGCCTGGAATGTTGGGACGTGCTCGTTCATGCTATGGACTGCTGCAGCTTGTCTCAGTTGGTTCGTCAATTCGATAGTTTGGAAGGACAACTTCATCAACCATGCGCCGGT   200
929pr1   ATGGCCCAAGATCCGACTTACCCGCTCTACCCAGTCGCCTTCTTCATCTCCCTCATCCTCGTCTTGATCCCTCTGCCGTGGCATCTTCAGGCCTGGAATGTTGGGACGTGCTCGTTCATGCTATGGACTGCTGCAGCTTGTCTCAGTTGGTTCGTCAATTCGATAGTTTGGAAGGACAACTTCATCAACCATGCGCCGGT   200
Consensusatggcccaagatccgacttacccgctctacccagtcgccttcttcatctccctcatcctcgtcttgatccctctgccgtggcatcttcaggcctggaatgttgggacgtgctcgttcatgctatggactgctgcagcttgtctcagttggttcgtcaattcgatagtttggaaggacaacttcatcaaccatgcgccggt

921pr1   TTGGTGCGATATTTCCTCGGCCCTCATAGTCGCGTACTCTGTGGCTCTTCCCGCGTCTTCCTTATGCATCACGCGACGACTCTTTAAGATTTCTCAAGTACAAACCGTCATGACCGGCCCGAAGGAGCGGCGAAAGGAACTTGCTATTGACTTGAGCCTCTGCGTGGGCTTGCCTGTGATTGTTCTGATCTTGCACTATT   400
929pr1   TTGGTGCGATATTTCCTCGGCCCTCATAGTCGCGTACTCTGTGGCTCTTCCCGCGTCTTCCTTATGCATCACGCGACGACTCTTTAAGATTTCTCAAGTACAAACCGTCATGACCGGCCCGAAGGAGCGGCGAAAGGAACTTGCTATTGACTTGAGCCTCTGCGTGGGCTTGCCTGTGATTGTTCTGATCTTGCACTATT   400
Consensusttggtgcgatatttcctcggccctcatagtcgcgtactctgtggctcttcccgcgtcttccttatgcatcacgcgacgactctttaagatttctcaagtacaaaccgtcatgaccggcccgaaggagcggcgaaaggaacttgctattgacttgagcctctgcgtgggcttgcctgtgattgttctgatcttgcactatt

921pr1   TCGTACAGGGACACCGGTTCGACATCTTCGAGCAAATCGGATGTTACCCTGTCACAGTCAACACGCCGCTGGCGTACCCGTTTGTGTGGATGTGGCCGTTACTTCTCGGGACGATCTCCATGATCTACTGCATCTTCACCGTCACATCCTTCCTGAAGCGCCGAAGAGAGATGAGCCAATTTCTCAGCAGCAATTCGCAA   600
929pr1   TCGTACAGGGACACCGGTTCGACATCTTCGAGCAAATCGGATGTTACCCTGTCACAGTCAACACGCCGCTGGCGTACCCGTTTGTGTGGATGTGGCCGTTACTTCTCGGGACGATCTCCATGATCTACTGCATCTTCACCGTCACATCCTTCCTGAAGCGCCGAAGAGAGATGAGCCAATTTCTCAGCAGCAATTCGCAA   600
Consensustcgtacagggacaccggttcgacatcttcgagcaaatcggatgttaccctgtcacagtcaacacgccgctggcgtacccgtttgtgtggatgtggccgttacttctcgggacgatctccatgatctactgcatcttcaccgtcacatccttcctgaagcgccgaagagagatgagccaatttctcagcagcaattcgcaa

921pr1   ATCACTTTCTCCCGATACTTCCGTCTTATGGCCTTGGCAACGATGGATACCCTGTTTACGGTCCCGCTTGCAACACTGGTGATAGTCCTGAACGCGACGGTTAACCCAATATATACTTGGAAGGGTCTTGATGACATCCACTGGGGTTTCAGTCGCGTTGAGACGGTCCCAGCGGTGTTTTGGCAGACCAACAGGTGGAC   800
929pr1   ATCACTTTCTCCCGATACTTCCGTCTTATGGCCTTGGCAACGATGGATACCCTGTTTACGGTCCCGCTTGCAACACTGGTGATAGTCCTGAACGCGACGGTTAACCCAATATATACTTGGAAGGGTCTTGATGACATCCACTGGGGTTTCAGTCGCGTTGAGACGGTCCCAGCGGTGTTTTGGCAGACCAACAGGTGGAC   800
Consensusatcactttctcccgatacttccgtcttatggccttggcaacgatggataccctgtttacggtcccgcttgcaacactggtgatagtcctgaacgcgacggttaacccaatatatacttggaagggtcttgatgacatccactggggtttcagtcgcgttgagacggtcccagcggtgttttggcagaccaacaggtggac

921pr1   CGTTGTCTCCTTCACGCTGAGCCGCGTATTCATCATCCTGTGTGCCGTTGTATTCTTCGTGTTCTTCGGGTTCGCAGAGGAGAATCGCAAGAACTACGCAAGGCTATACTGGAAGATCATGAAACGATTTGGTTACCAGCCAAAGCCCAAGGGGACGACGTTCACGAATTCGTCAGCTGTGCGGACACCGCATCTTCCAG   1000
929pr1   CGTTGTCTCCTTCACGCTGAGCCGCGTATTCATCATCCTGTGTGCCGTTGTATTCTTCGTGTTCTTCGGGTTCGCAGAGGAGAATCGCAAGAACTACGCAAGGCTATACTGGAAGATCATGAAACGATTTGGTTACCAGCCAAAGCCCAAGGGGACGACGTTCACGAATTCGTCAGCTGTGCGGACACCGCATCTTCCAG   1000
Consensuscgttgtctccttcacgctgagccgcgtattcatcatcctgtgtgccgttgtattcttcgtgttcttcgggttcgcagaggagaatcgcaagaactacgcaaggctatactggaagatcatgaaacgatttggttaccagccaaagcccaaggggacgacgttcacgaattcgtcagctgtgcggacaccgcatcttccag

921pr1   TCATGTCCGGTGGAGGCCAACATGGACTCAGGGTTCTTGTCAGCACAGATGCGAGAGCCGAGAAACGGGATTCGTTCATTTCATCGATTGGAGACCTTTCATCGTCGATATCTGTGAATTCGACGTATGACGAGCGAAAAGACTTTAGGTCGCCGATTGAGACGACGAGAACGGATCGGGACAGCCTACCACCGTCACCC   1200
929pr1   TCATGTCCGGTGGAGGCCAACATGGACTCAGGGTTCTTGTCAGCACAGATGCGAGAGCCGAGAAACGGGATTCGTTCATTTCATCGATTGGAGACCTTTCATCGTCGATATCTGTGAATTCGACGTATGACGAGCGAAAAGACTTTAGGTCGCCGATTGAGACGACGAGAACGGATCGGGACAGCCTACCACCGTCACCC   1200
Consensustcatgtccggtggaggccaacatggactcagggttcttgtcagcacagatgcgagagccgagaaacgggattcgttcatttcatcgattggagacctttcatcgtcgatatctgtgaattcgacgtatgacgagcgaaaagactttaggtcgccgattgagacgacgagaacggatcgggacagcctaccaccgtcaccc

921pr1   GATGATATAGAACTTCCGAACTTACATCATGTTCTTGCTACTACAACGACGACAACGACGGGGGCAGTAACATCGACACAACCGCCGGCTCGACCAGCGCGGCCGACCTCGCTAGGAGACGTTGTATA   1328
929pr1   GATGATATAGAACTTCCGAACTTACATCATGTTCTTGCTACTACAACGACGACAACGACGGGGGCAGTAACATCGACACAACCGCCGGCTCGACCAGCGCGGCCGACCTCGCTAGGAGACGTTGTATA   1328
Consensusgatgatatagaacttccgaacttacatcatgttcttgctactacaacgacgacaacgacgggggcagtaacatcgacacaaccgccggctcgaccagcgcggccgacctcgctaggagacgttgtata

921pr2   ATGTCTGCCTTCAGCATATTCTACTCGACACTCACCTTCTTTTCTTCCGCTCTCGCTTTATTTCTGCTCCCTTGGTTCGTTCGTTTTCGAAGTATACCTGGGCTGTTTGCGATTGGATGGCTTTTTGTGGTTAACCTCTTGTACGGGTTAAATGCGGCTGTTTTTACTGGTGATGGTAGCGGGGTCGGTGCTGGTGGGG   200
929pr2   ATGTCTGCCTTCAGCATATTCTACTCGACACTCACCTTCTTTTCTTCCGCTCTCGCTTTATTTCTGCTCCCTTGGTTCGTTCGTTTTCGAAGTATACCTGGGCTGTTTGCGATTGGATGGCTTTTTGTGGTTAACCTCTTGTACGGGTTAAATGCGGCTGTTTTTACTGGTGATGGTAGCGGGGTCGGTGCTGGTGGGG   200
Consensusatgtctgccttcagcatattctactcgacactcaccttcttttcttccgctctcgctttatttctgctcccttggttcgttcgttttcgaagtatacctgggctgtttgcgattggatggctttttgtggttaacctcttgtacgggttaaatgcggctgtttttactggtgatggtagcggggtcggtgctggtgggg

921pr2   GGCATCTAGGGCGGGCGATGAGAATATGCCGGTGCTGTTGAAGTTCCCTGCGTATTGCGATATTGTCACGAAGCTCCTTGTGGGGATGCAGATGGCCGTACCGACTGCCATGCTCTGCTATACCTTCTCCGTACATGATTGGATGGTCCGTCAACGGGAATGTGATACGAGCACGAAGCGCCTCGAATACAGAAGCCACT   400
929pr2   GGCATCTAGGGCGGGCGATGAGAATATGCCGGTGCTGTTGAAGTTCCCTGCGTATTGCGATATTGTCACGAAGCTCCTTGTGGGGATGCAGATGGCCGTACCGACTGCCATGCTCTGCTATACCTTCTCCGTACATGATTGGATGGTCCGTCAACGGGAATGTGATACGAGCACGAAGCGCCTCGAATACAGAAGCCACT   400
Consensusggcatctagggcgggcgatgagaatatgccggtgctgttgaagttccctgcgtattgcgatattgtcacgaagctccttgtggggatgcagatggccgtaccgactgccatgctctgctataccttctccgtacatgattggatggtccgtcaacgggaatgtgatacgagcacgaagcgcctcgaatacagaagccact

921pr2   TTCACGTCGCACTCTGTCTCCTTGCGCCGCTCGTATACCTGTCTCTTCACATCGCAGCGCAGGGGCATCGCTTCGATATAGTCGACAACGTCGGGTGTATTCCGTCGTCGTCGAGCACCGCATTGGGTTGGGTGCTCATTTGGCAGCTCCTTCTCGTTCCCTTCGCTGCTATACTCGTATACGGCTATTTCGTTTTCAGG   600
929pr2   TTCACGTCGCACTCTGTCTCCTTGCGCCGCTCGTATACCTGTCTCTTCACATCGCAGCGCAGGGGCATCGCTTCGATATAGTCGACAACGTCGGGTGTATTCCGTCGTCGTCGAGCACCGCATTGGGTTGGGTGCTCATTTGGCAGCTCCTTCTCGTTCCCTTCGCTGCTATACTCGTATACGGCTATTTCGTTTTCAGG   600
Consensusttcacgtcgcactctgtctccttgcgccgctcgtatacctgtctcttcacatcgcagcgcaggggcatcgcttcgatatagtcgacaacgtcgggtgtattccgtcgtcgtcgagcaccgcattgggttgggtgctcatttggcagctccttctcgttcccttcgctgctatactcgtatacggctatttcgttttcagg

921pr2   TATTCTTGGTCGCATTATCGCAGATACTGCGCGGAATACCTGACAACCTGGGAATTGGTCGACTCCGATCTGCTTTTCACGAACGCGCCGTTTGGGATCACACGCATTGTTCGGTTGTCTGCGATGATTGTCGCTGTAATGCTCTGTACTCTCGGTGCTCCGTATTACGCGCTCTCCGTGTTATCTCAAGCCGACGACAT   800
929pr2   TATTCTTGGTCGCATTATCGCAGATACTGCGCGGAATACCTGACAACCTGGGAATTGGTCGACTCCGATCTGCTTTTCACGAACGCGCCGTTTGGGATCACACGCATTGTTCGGTTGTCTGCGATGATTGTCGCTGTAATGCTCTGTACTCTCGGTGCTCCGTATTACGCGCTCTCCGTGTTATCTCAAGCCGACGACAT   800
Consensustattcttggtcgcattatcgcagatactgcgcggaatacctgacaacctgggaattggtcgactccgatctgcttttcacgaacgcgccgtttgggatcacacgcattgttcggttgtctgcgatgattgtcgctgtaatgctctgtactctcggtgctccgtattacgcgctctccgtgttatctcaagccgacgacat

921pr2   CGCGATTGCTAGAAGACCAGATTTCTATCGTGTCGAAGTCTTTAGCTCGCTGAACGATGCGTCTCCCAGCCTGACGAGCATGCGACTATTCCTGTGGTCAGGTCCGCTATATAGCATCCTCCTCTTCGCCGCTAGCGTGGATAAATCTGTCCTGCAATCATATCCCTTCATGATCAGGAAATATATCCTGCGACAGCGCG   1000
929pr2   CGCGATTGCTAGAAGACCAGATTTCTATCGTGTCGAAGTCTTTAGCTCGCTGAACGATGCGTCTCCCAGCCTGACGAGCATGCGACTATTCCTGTGGTCAGGTCCGCTATATAGCATCCTCCTCTTCGCCGCTAGCGTGGATAAATCTGTCCTGCAATCATATCCCTTCATGATCAGGAAATATATCCTGCGACAGCGCG   1000
Consensuscgcgattgctagaagaccagatttctatcgtgtcgaagtctttagctcgctgaacgatgcgtctcccagcctgacgagcatgcgactattcctgtggtcaggtccgctatatagcatcctcctcttcgccgctagcgtggataaatctgtcctgcaatcatatcccttcatgatcaggaaatatatcctgcgacagcgcg

921pr2   TCGGAGCGAACCCCGACCCGAGTCCAGGGATTGACCTCTCAACTCTCACACCAATCCCTGTACGGAGGCGAACTCACAGGCGTGTCATACGCAAGTCGCGCCAATACGACCCACCCCTGTCGGCGCTGGATTTCGATATCCCGCTTCCTCCAAGGCGTGATCCCGGATTCCTTAAGACTGCTTCACTTCCTCCTGTCCCG   1200
929pr2   TCGGAGCGAACCCCGACCCGAGTCCAGGGATTGACCTCTCAACTCTCACACCAATCCCTGTACGGAGGCGAACTCACAGGCGTGTCATACGCAAGTCGCGCCAATACGACCCACCCCTGTCGGCGCTGGATTTCGATATCCCGCTTCCTCCAAGGCGTGATCCCGGATTCCTTAAGACTGCTTCACTTCCTCCTGTCCCG   1200
Consensustcggagcgaaccccgacccgagtccagggattgacctctcaactctcacaccaatccctgtacggaggcgaactcacaggcgtgtcatacgcaagtcgcgccaatacgacccacccctgtcggcgctggatttcgatatcccgcttcctccaaggcgtgatcccggattccttaagactgcttcacttcctcctgtcccg

921pr2   AATACGCCTTTGGCCCCTGTCCGGGGCCCTCGAGAGCCGGAAATAGCGTACGTAAAGGACGTGGACGGTACCACTCGACGTATTCGGAATGCAAGAAGTGGCGAGTCTTTGGGTGCGATGCATTTACATGCACAGACGAACAGCAGATTTCCATTGTCCACTATTCTGGAAATACCGCCTATCCGTGAAAATAGCGGATG   1400
929pr2   AATACGCCTTTGGCCCCTGTCCGGGGCCCTCGAGAGCCGGAAATAGCGTACGTAAAGGACGTGGACGGTACCACTCGACGTATTCGGAATGCAAGAAGTGGCGAGTCTTTGGGTGCGATGCATTTACATGCACAGACGAACAGCAGATTTCCATTGTCCACTATTCTGGAAATACCGCCTATCCGTGAAAATAGCGGATG   1400
Consensusaatacgcctttggcccctgtccggggccctcgagagccggaaatagcgtacgtaaaggacgtggacggtaccactcgacgtattcggaatgcaagaagtggcgagtctttgggtgcgatgcatttacatgcacagacgaacagcagatttccattgtccactattctggaaataccgcctatccgtgaaaatagcggatg
```

```
921pr3    ATGATGGACGCGTATGACCCTCGCACGCATCCTCCCCCACACGTCCAATACGGCCAACACTCCTACGCTTCCTCGTACTACGCTCCTGACATCCACTCGTCGGCCTCCACCTCTTCATCTGCGTACCCGTCCCCTGCCTACCCTTCGTACGCCTACCAATCAGCATCTCCCTACAGTAGTGGTAACTCCGGGTACACCGC   200
929pr3    ATGATGGACGCGTATGACCCTCGCACGCATCCTCCCCCACACGTCCAATACGGCCAACACTCCTACGCTTCCTCGTACTACGCTCCTGACATCCACTCGTCGGCCTCCACCTCTTCATCTGCGTACCCGTCCCCTGCCTACCCTTCGTACGCCTACCAATCAGCATCTCCCTACAGTAGTGGTAACTCCGGGTACACCGC   200
Consensusatgatggacgcgtatgaccctcgcacgcatcctcccccacacgtccaatacggccaacactcctacgcttcctcgtactacgctcctgacatccactcgtcggcctccacctcttcatctgcgtacccgtcccctgcctacccttcgtacgcctaccaatcagcatctccctacagtagtggtaactccgggtacaccgc

921pr3    AGTTGAGTCGCAGACGTTGGGTTACTCCGTGCCATCCCAGAGTGCCGAGCCTTCCCTACCTAGTACTTTAACAGCGAGGGAAGGCGATCGCAGTATGGCAAGTAGCGCGCGCTCCTCCCCGGACGCAGGTCCTTCTCTTGACTATTCGTCAATGTCTTCCTCCGCTCCTAACCACCATCAGTCGCGTCCAATGGTGCACT   400
929pr3    AGTTGAGTCGCAGACGTTGGGTTACTCCGTGCCATCCCAGAGTGCCGAGCCTTCCCTACCTAGTACTTTAACAGCGAGGGAAGGCGATCGCAGTATGGCAAGTAGCGCGCGCTCCTCCCCGGACGCAGGTCCTTCTCTTGACTATTCGTCAATGTCTTCCTCCGCTCCTAACCACCATCAGTCGCGTCCAATGGTGCACT   400
Consensusagttgagtcgcagacgttgggttactccgtgccatcccagagtgccgagccttccctacctagtactttaacagcgagggaaggcgatcgcagtatggcaagtagcgcgcgctcctccccggacgcaggtccttctcttgactattcgtcaatgtcttcctccgctcctaaccaccatcagtcgcgtccaatggtgcact

921pr3    CTCAGTCGCACTCGCCCAATGCCTCAACATCCAGCAGCGCCAGTTCCTCACCTCGTCCCTCACAGCAGCCTCAGATCAGTTCTCTCCCCATAGCCCCCTCCGCCCTCCCTCATAACTCGGCTGGGTCCCATAACCAGATCACGTTCAATGGGACACCGCTCTCACGGCCTCTGACTCACAAGGAGCAGGAGATGCTCGCA   600
929pr3    CTCAGTCGCACTCGCCCAATGCCTCAACATCCAGCAGCGCCAGTTCCTCACCTCGTCCCTCACAGCAGCCTCAGATCAGTTCTCTCCCCATAGCCCCCTCCGCCCTCCCTCATAACTCGGCTGGGTCCCATAACCAGATCACGTTCAATGGGACACCGCTCTCACGGCCTCTGACTCACAAGGAGCAGGAGATGCTCGCA   600
Consensusctcagtcgcactcgcccaatgcctcaacatccagcagcgccagttcctcacctcgtccctcacagcagcctcagatcagttctctccccatagccccctccgccctccctcataactcggctgggtcccataaccagatcacgttcaatgggacaccgctctcacggcctctgactcacaaggagcaggagatgctcgca

921pr3    CATCTTGATCGCCTCAAGTTCTTCCTCGCGACTGCTCCGAGCCGCTGGGCAACAGCGGACGGCGACGGGCAAGGAACACCGATGATGCCTCACCCAAATACTCACCCCGCACTGAACCGCTTCTTGTTGCCGTCTGGTGAATACGTGACCTGTGTGCTCTGGTCAGGGCTGTACCATATCACTGGAACTGACATTGTCCG   800
929pr3    CATCTTGATCGCCTCAAGTTCTTCCTCGCGACTGCTCCGAGCCGCTGGGCAACAGCGGACGGCGACGGGCAAGGAACACCGATGATGCCTCACCCAAATACTCACCCCGCACTGAACCGCTTCTTGTTGCCGTCTGGTGAATACGTGACCTGTGTGCTCTGGTCAGGGCTGTACCATATCACTGGAACTGACATTGTCCG   800
Consensuscatcttgatcgcctcaagttcttcctcgcgactgctccgagccgctgggcaacagcggacggcgacgggcaaggaacaccgatgatgcctcacccaaatactcaccccgcactgaaccgcttcttgttgccgtctggtgaatacgtgacctgtgtgctctggtcagggctgtaccatatcactggaactgacattgtccg

921pr3    CGCTCTCGTATTTCGATTCGAGGCTTTCGGTCGTCCTGTACGCAACATGAAGAAGTTCGAGGAAGGCGTATTCTCCGACCTCCGTAACTTGAAGCCTGGGACAGACGCCTGCCTTGAAGAGCCAAAGTCCCCATTCCTCGATCTTCTGTTCAAGTATCAATGCATCAGGACGCAGAAGAAGCAGAAGGTCTTCTATTGGC   1000
929pr3    CGCTCTCGTATTTCGATTCGAGGCTTTCGGTCGTCCTGTACGCAACATGAAGAAGTTCGAGGAAGGCGTATTCTCCGACCTCCGTAACTTGAAGCCTGGGACAGACGCCTGCCTTGAAGAGCCAAAGTCCCCATTCCTCGATCTTCTGTTCAAGTATCAATGCATCAGGACGCAGAAGAAGCAGAAGGTCTTCTATTGGC   1000
Consensuscgctctcgtatttcgattcgaggctttcggtcgtcctgtacgcaacatgaagaagttcgaggaaggcgtattctccgacctccgtaacttgaagcctgggacagacgcctgccttgaagagccaaagtccccattcctcgatcttctgttcaagtatcaatgcatcaggacgcagaagaagcagaaggtcttctattggc

921pr3    TGTTCCTGGATGCCCTTGAGCGTGATTTGAAGCGCGAGAAGATGGGCCTGGAACCGACAACGGTTGTAGTCGGCGAACCTGCTCTGTCGTTTAGGTACGACAGTAAGCGCTCTCTATACGAGCAGTTCTCGAAAGCGCAAGGCATTCAAGATGGCGAAGGTGAACTGGAATCCGCTGTTCGTCGTGCGGAAGAGGCAATG   1200
929pr3    TGTTCCTGGATGCCCTTGAGCGTGATTTGAAGCGCGAGAAGATGGGCCTGGAACCGACAACGGTTGTAGTCGGCGAACCTGCTCTGTCGTTTAGGTACGACAGTAAGCGCTCTCTATACGAGCAGTTCTCGAAAGCGCAAGGCATTCAAGATGGCGAAGGTGAACTGGAATCCGCTGTTCGTCGTGCGGAAGAGGCAATG   1200
Consensustgttcctggatgcccttgagcgtgatttgaagcgcgagaagatgggcctggaaccgacaacggttgtagtcggcgaacctgctctgtcgtttaggtacgacagtaagcgctctctatacgagcagttctcgaaagcgcaaggcattcaagatggcgaaggtgaactggaatccgctgttcgtcgtgcggaagaggcaatg

921pr3    GCTATGGAGCAAGAGAGCGAAGCTCGAAGAGTGGCCTCAGGATCTACTGGGAGCTCTGAGCGTGATGTTGCACCCAGCACTATCTTCAACATGTTCTCACTGTTTGAGGGTAGCCCAAACTACAAGCAACGCCGCAAGAAGGGTCCGCGCACGGGACGCAGTGGCCTTGGACGTGATTCTGCATCGGAGGAAGAGCGCGA   1400
929pr3    GCTATGGAGCAAGAGAGCGAAGCTCGAAGAGTGGCCTCAGGATCTACTGGGAGCTCTGAGCGTGATGTTGCACCCAGCACTATCTTCAACATGTTCTCACTGTTTGAGGGTAGCCCAAACTACAAGCAACGCCGCAAGAAGGGTCCGCGCACGGGACGCAGTGGCCTTGGACGTGATTCTGCATCGGAGGAAGAGCGCGA   1400
Consensusgctatggagcaagagagcgaagctcgaagagtggcctcaggatctactgggagctctgagcgtgatgttgcacccagcactatcttcaacatgttctcactgtttgagggtagcccaaactacaagcaacgccgcaagaagggtccgcgcacgggacgcagtggccttggacgtgattctgcatcggaggaagagcgcga

921pr3    AATGCACGGCGGTGCTGGATCAGGGGATGTCGTAGAAGATGTCAACGGCGAGATGAGCGCAGCTGCAATGTTTGACGCTCAGGCCGGTCTCGGTGGCCATCAAGCGAGAGCACAACGTCAAGCCGAGAGTCAGCGTGCTATCCAAGCGCAGCGTCTCGCAATCATCAACCAGCAAACGAATTCTATCTCCAAGACGCGAC   1600
929pr3    AATGCACGGCGGTGCTGGATCAGGGGATGTCGTAGAAGATGTCAACGGCGAGATGAGCGCAGCTGCAATGTTTGACGCTCAGGCCGGTCTCGGTGGCCATCAAGCGAGAGCACAACGTCAAGCCGAGAGTCAGCGTGCTATCCAAGCGCAGCGTCTCGCAATCATCAACCAGCAAACGAATTCTATCTCCAAGACGCGAC   1600
Consensusaatgcacggcggtgctggatcaggggatgtcgtagaagatgtcaacggcgagatgagcgcagctgcaatgtttgacgctcaggccggtctcggtggccatcaagcgagagcacaacgtcaagccgagagtcagcgtgctatccaagcgcagcgtctcgcaatcatcaaccagcaaacgaattctatctccaagacgcgac

921pr3    AATTCCCTGGTCGTGCAATGCACCCATCGTTAAGCAGTTCACAATTGCACAGCAACTCGCTTCCAGCGAACTTTGACACAGGACGCAAGACGAGGGCGTATGTTTGTCCGCTGTACTCATGCCAGCGCCTGTTCAAGCGCCTCGAGCATTTGAAGCGGCACGTTCGGATGCACACGATGGAGCGGCCACACCAGTGCGAA   1800
929pr3    AATTCCCTGGTCGTGCAATGCACCCATCGTTAAGCAGTTCACAATTGCACAGCAACTCGCTTCCAGCGAACTTTGACACAGGACGCAAGACGAGGGCGTATGTTTGTCCGCTGTACTCATGCCAGCGCCTGTTCAAGCGCCTCGAGCATTTGAAGCGGCACGTTCGGATGCACACGATGGAGCGGCCACACCAGTGCGAA   1800
Consensusaattccctggtcgtgcaatgcacccatcgttaagcagttcacaattgcacagcaactcgcttccagcgaactttgacacaggacgcaagacgagggcgtatgtttgtccgctgtactcatgccagcgcctgttcaagcgcctcgagcatttgaagcggcacgttcggatgcacacgatggagcggccacaccagtgcgaa

921pr3    GTGTGCCAGCGCCGATTCTCGCGAGCTGATAATCTCAACCAGCACATGCGCACCCACTCGCGCGGTGGTAACGTCAACATGTTGCCAGTTGGTGATATAGACGGGGATGAGGGTGTTGATCAAGTGATGTACGCACCTGGCGAGCAGTACGACCTGAATGCATGCGAAATTGAGGCGGATGCATCGATGCGTTTTGAGGA   2000
929pr3    GTGTGCCAGCGCCGATTCTCGCGAGCTGATAATCTCAACCAGCACATGCGCACCCACTCGCGCGGTGGTAACGTCAACATGTTGCCAGTTGGTGATATAGACGGGGATGAGGGTGTTGATCAAGTGATGTACGCACCTGGCGAGCAGTACGACCTGAATGCATGCGAAATTGAGGCGGATGCATCGATGCGTTTTGAGGA   2000
Consensusgtgtgccagcgccgattctcgcgagctgataatctcaaccagcacatgcgcacccactcgcgcggtggtaacgtcaacatgttgccagttggtgatatagacggggatgagggtgttgatcaagtgatgtacgcacctggcgagcagtacgacctgaatgcatgcgaaattgaggcggatgcatcgatgcgttttgagga

921pr3    CGACGAGTATACAGGCTCTTATGTTGCGCCCTCATCGGCACCCAACGGCTCTCCCTACAGTAGTTTCGTTAGCGCAGAGAACTCTCAGTTCACTAATTTGGTGACGAGTCCTGAAAATTCGCCTCGGTTGACTGAGAGCAACTGGTCGGCGACACACGGTCATTCAAACTCGTTGCCAGCTGGCTACGACTACGGATCTT   2200
929pr3    CGACGAGTATACAGGCTCTTATGTTGCGCCCTCATCGGCACCCAACGGCTCTCCCTACAGTAGTTTCGTTAGCGCAGAGAACTCTCAGTTCACTAATTTGGTGACGAGTCCTGAAAATTCGCCTCGGTTGACTGAGAGCAACTGGTCGGCGACACACGGTCATTCAAACTCGTTGCCAGCTGGCTACGACTACGGATCTT   2200
Consensuscgacgagtatacaggctcttatgttgcgccctcatcggcacccaacggctctccctacagtagtttcgttagcgcagagaactctcagttcactaatttggtgacgagtcctgaaaattcgcctcggttgactgagagcaactggtcggcgacacacggtcattcaaactcgttgccagctggctacgactacggatctt

921pr3    CGCATCCGTCACCGGCGTTCAGTACCTCCAGCGCGCCGTCTTCACGGTTGGCGACTGCGTACAGCAATCCAACGTACTCTGTCCCACACGAGGTCCCCTCCAACTCCTCATCGTATGCCGCTTCTTCGGCCGGCTCGCTTTCGGCGCCTGCGCATAAGCAATCGTTCGACCACGCCGCACTGTTGGCCCCATCACTTGGA   2400
929pr3    CGCATCCGTCACCGGCGTTCAGTACCTCCAGCGCGCCGTCTTCACGGTTGGCGACTGCGTACAGCAATCCAACGTACTCTGTCCCACACGAGGTCCCCTCCAACTCCTCATCGTATGCCGCTTCTTCGGCCGGCTCGCTTTCGGCGCCTGCGCATAAGCAATCGTTCGACCACGCCGCACTGTTGGCCCCATCACTTGGA   2400
Consensuscgcatccgtcaccggcgttcagtacctccagcgcgccgtcttcacggttggcgactgcgtacagcaatccaacgtactctgtcccacacgaggtcccctccaactcctcatcgtatgccgcttcttcggccggctcgctttcggcgcctgcgcataagcaatcgttcgaccacgccgcactgttggccccatcacttgga

921pr3    AGTGTGCAGGGCTTCTCTACAAGTGCCGTCGGTCCGGTGCGCCGTTACCGCAGCGTCACCCCGACCATTGCACGTACCGGGGAGCAGATCCGTCGTCCACTAACTGCGAATACGCTTGACGCAAGTCCGCTCGGACCGGGTGCCGTGACTTCGCGCGGTTACCATCCCTATTCTTACGCTTCTGTTTCTCAGCACTCGAG   2600
929pr3    AGTGTGCAGGGCTTCTCTACAAGTGCCGTCGGTCCGGTGCGCCGTTACCGCAGCGTCACCCCGACCATTGCACGTACCGGGGAGCAGATCCGTCGTCCACTAACTGCGAATACGCTTGACGCAAGTCCGCTCGGACCGGGTGCCGTGACTTCGCGCGGTTACCATCCCTATTCTTACGCTTCTGTTTCTCAGCACTCGAG   2600
Consensusagtgtgcagggcttctctacaagtgccgtcggtccggtgcgccgttaccgcagcgtcaccccgaccattgcacgtaccggggagcagatccgtcgtccactaactgcgaatacgcttgacgcaagtccgctcggaccgggtgccgtgacttcgcgcggttaccatccctattcttacgcttctgtttctcagcactcgag

921pr3    CCCAGCAGCGTACCAGACTCAACTTGACTATTCGCAGCACACACCCGGTACGCAGTCGAATTCACCCGCAGGCTCAACAGTCTACCAAGAGGACTTTCAAACGTTGGTTGGGCTAGATGCAGCGACTCTTGGCGGTTACGAGAGTAACTCGATTGCGGCATCGGGTGTTGCGGCGTACGCGAGTGATGGATATTCGGAAC   2800
929pr3    CCCAGCAGCGTACCAGACTCAACTTGACTATTCGCAGCACACACCCGGTACGCAGTCGAATTCACCCGCAGGCTCAACAGTCTACCAAGAGGACTTTCAAACGTTGGTTGGGCTAGATGCAGCGACTCTTGGCGGTTACGAGAGTAACTCGATTGCGGCATCGGGTGTTGCGGCGTACGCGAGTGATGGATATTCGGAAC   2800
Consensuscccagcagcgtaccagactcaacttgactattcgcagcacacacccggtacgcagtcgaattcacccgcaggctcaacagtctaccaagaggactttcaaacgttggttgggctagatgcagcgactcttggcggttacgagagtaactcgattgcggcatcgggtgttgcggcgtacgcgagtgatggatattcggaac

921pr3    CAGCGCCGCAGGGACAATTCACTGCCTCGGCGGATTCCGCAGCAGCGGCGACTAATTACTATGGGGATCTGGGCAATCTGAACGGGACCGCGCAATTCTCGGAACAGTCATACTCCTATGCGTCCTA   2927
929pr3    CAGCGCCGCAGGGACAATTCACTGCCTCGGCGGATTCCGCAGCAGCGGCGACTAATTACTATGGGGATCTGGGCAATCTGAACGGGACCGCGCAATTCTCGGAACAGTCATACTCCTATGCGTCCTA   2927
Consensuscagcgccgcagggacaattcactgcctcggcggattccgcagcagcggcgactaattactatggggatctgggcaatctgaacgggaccgcgcaattctcggaacagtcatactcctatgcgtccta
```

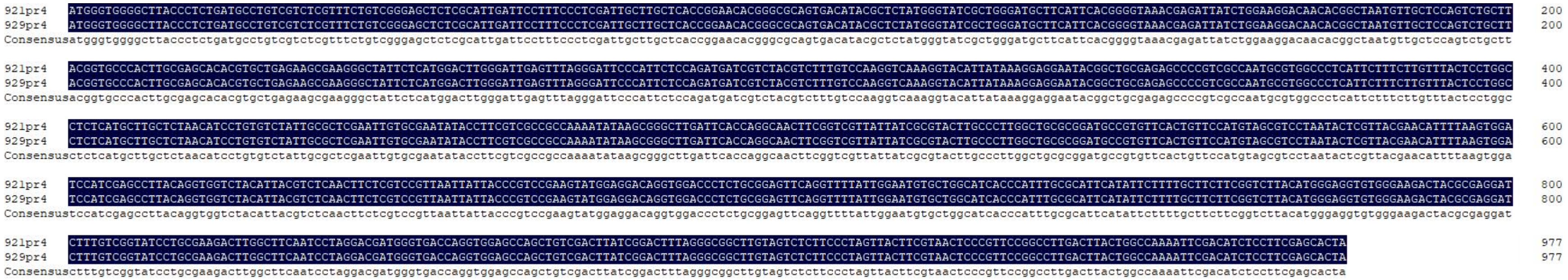

Fig. S1 Nucleotide sequence alignment of three *HD* and four *PR* genes between strains 921 and 929. The sequences labeled 921 in the sequence name were from strain 921, while labeled 929 in the sequence name were from strain 929.

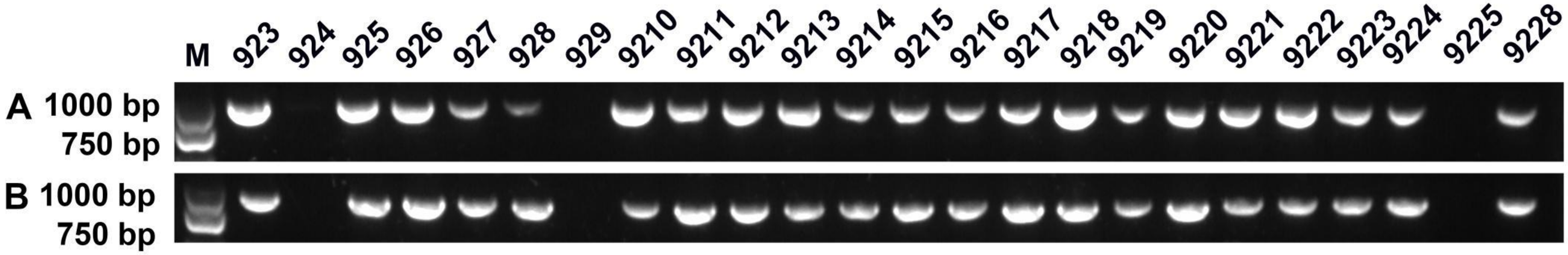

Fig. S2 PCR verification of the linkage of mating type genes *HD1* and *HD1.2*. **(A)** Strains that successfully amplified with primers A1-HD1F1/R1. **(B)** Strains that successfully amplified with primers A1-HD1.2F1/R1. M indicates the 2-kb size marker.

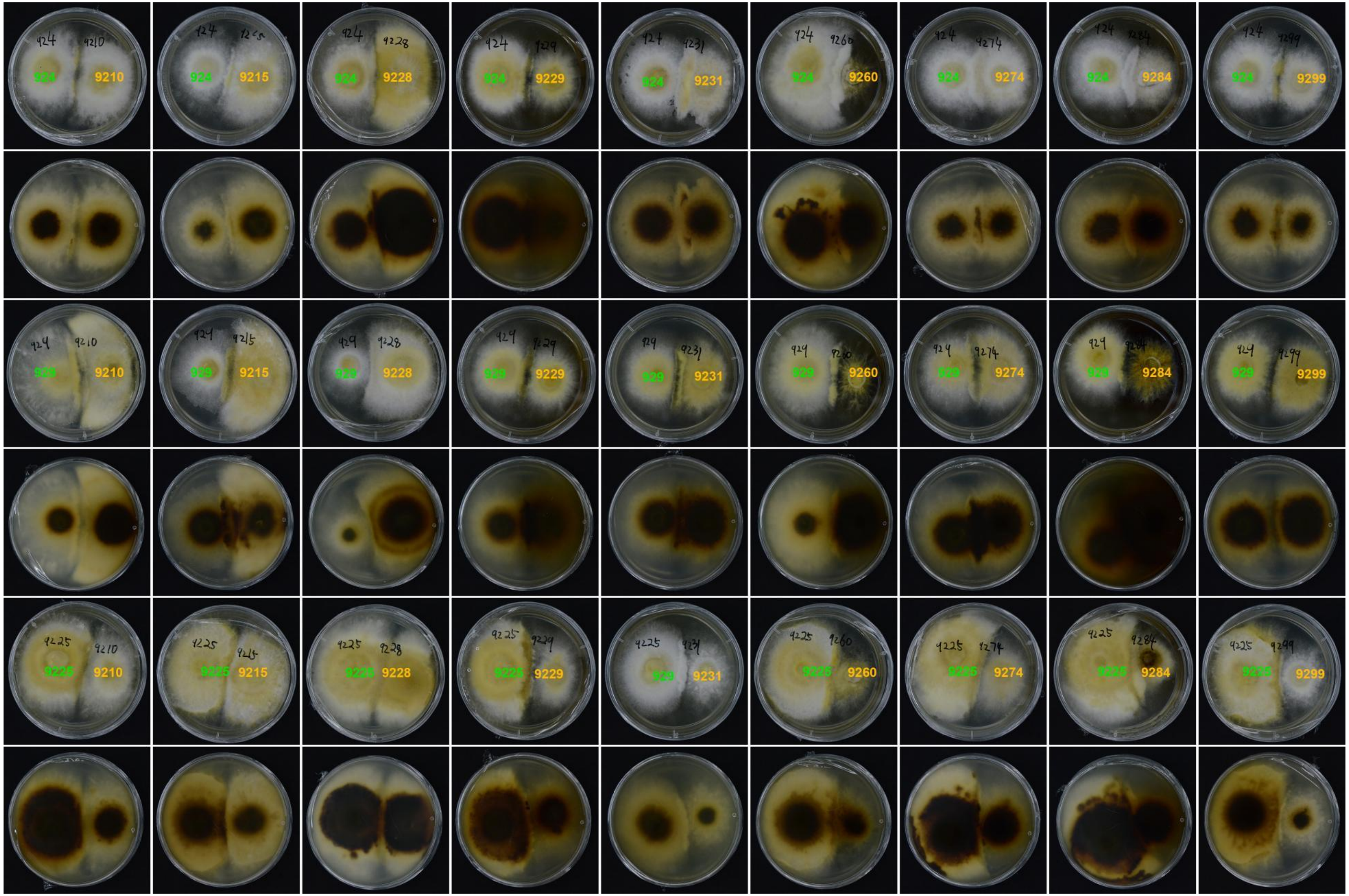
924 9210
924 9215
924 9228
924 9229
924 9231
924 9260
924 9274
924 9284
924 9299
929 9210
929 9215
929 9228
929 9229
929 9231
929 9260
929 9274
929 9284
929 9299
9225 9210
9225 9215
9225 9228
9225 9229
929 9231
9225 9260
9225 9274
9225 9284
9225 9299

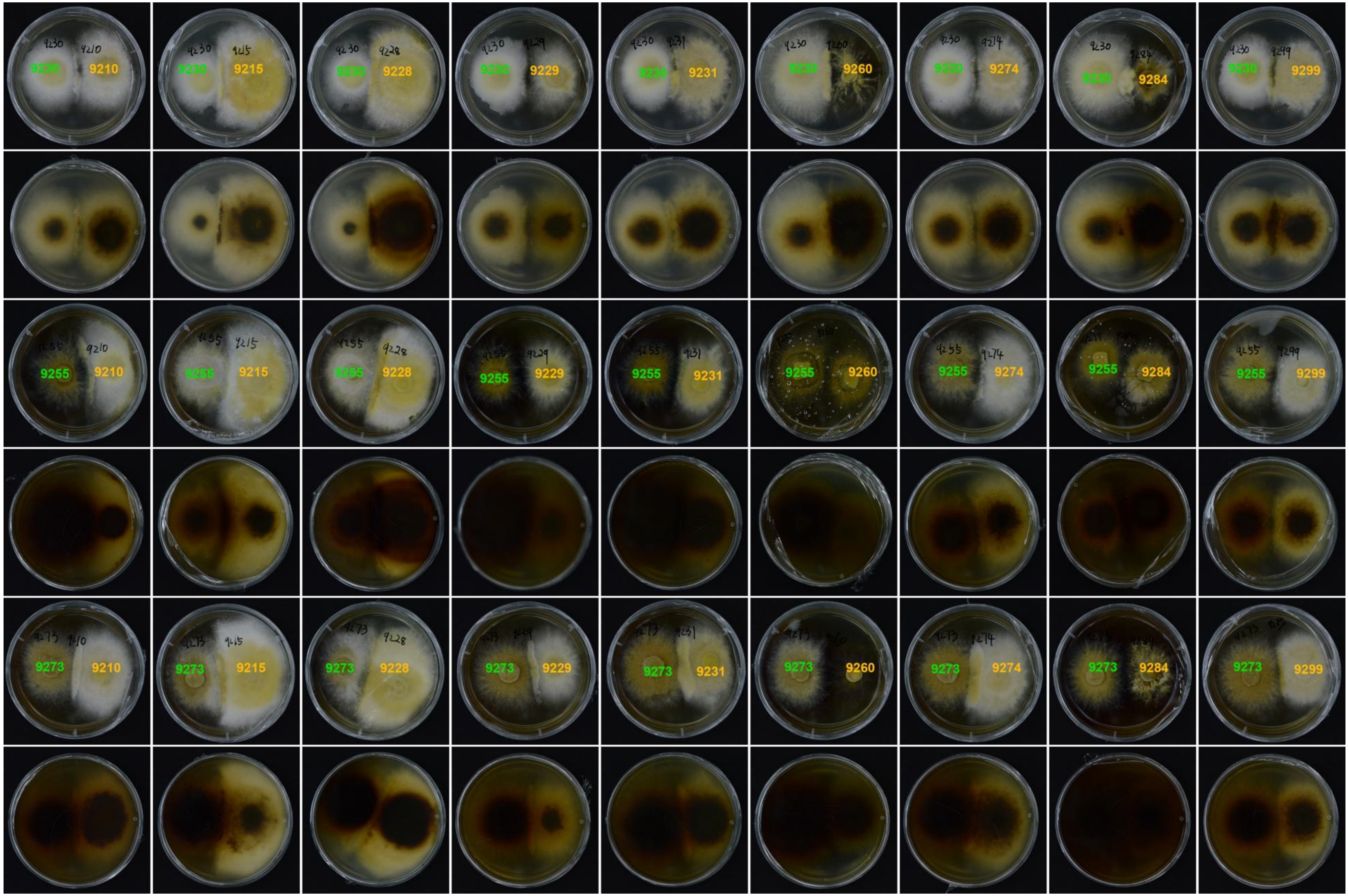

9230
9210
9230
9215
9230
9228
9230
9229
9230
9231
9230
9260
9230
9274
9230
9284
9230
9299
9255
9210
9255
9215
9255
9228
9255
9229
9255
9231
9255
9260
9255
9274
9255
9284
9255
9299
9273
9210
9273
9215
9273
9228
9273
9229
9273
9231
9273
9260
9273
9274
9273
9284
9273
9299

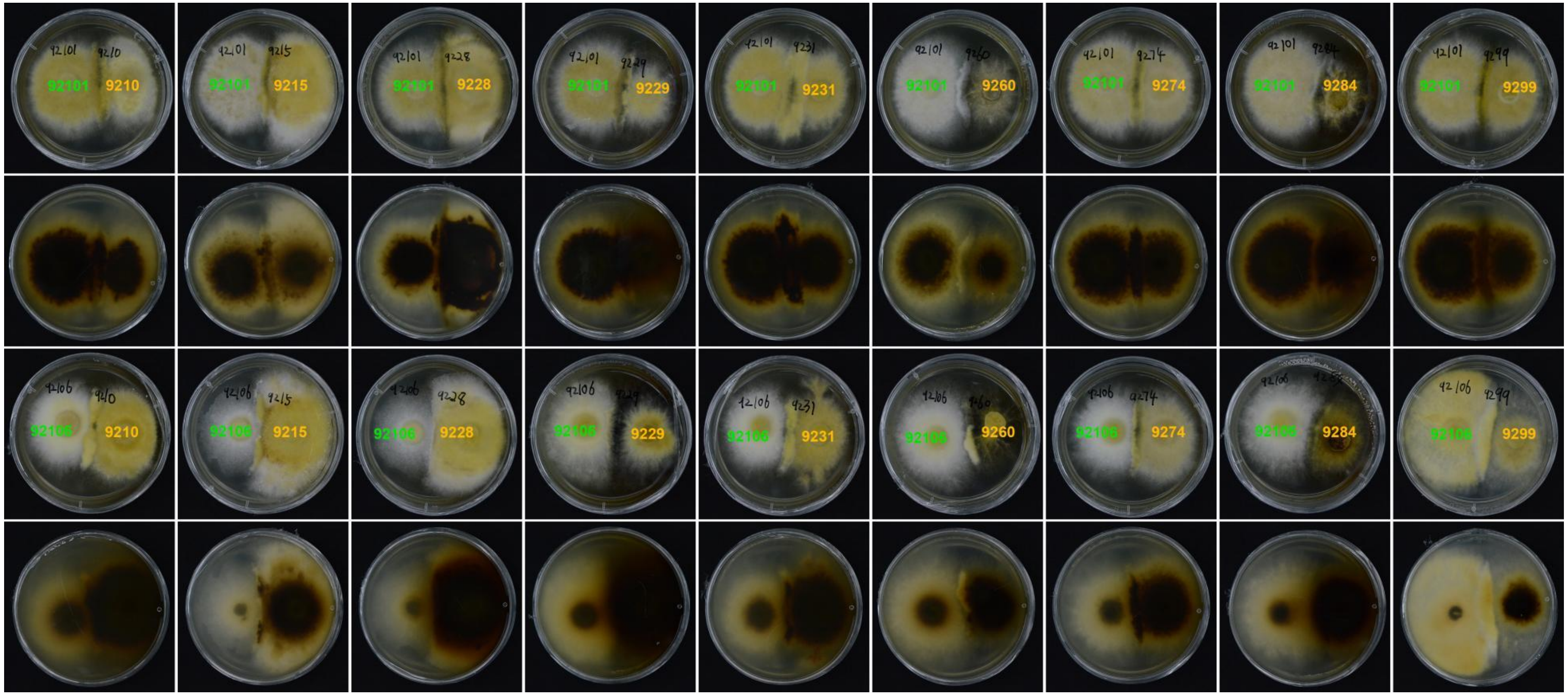

Fig. S3 Mating reactions of SSIs. Qrange labels indicate homokaryotic strains with mating type *A1*; green labels indicate homokaryotic strains with mating type *A2*.

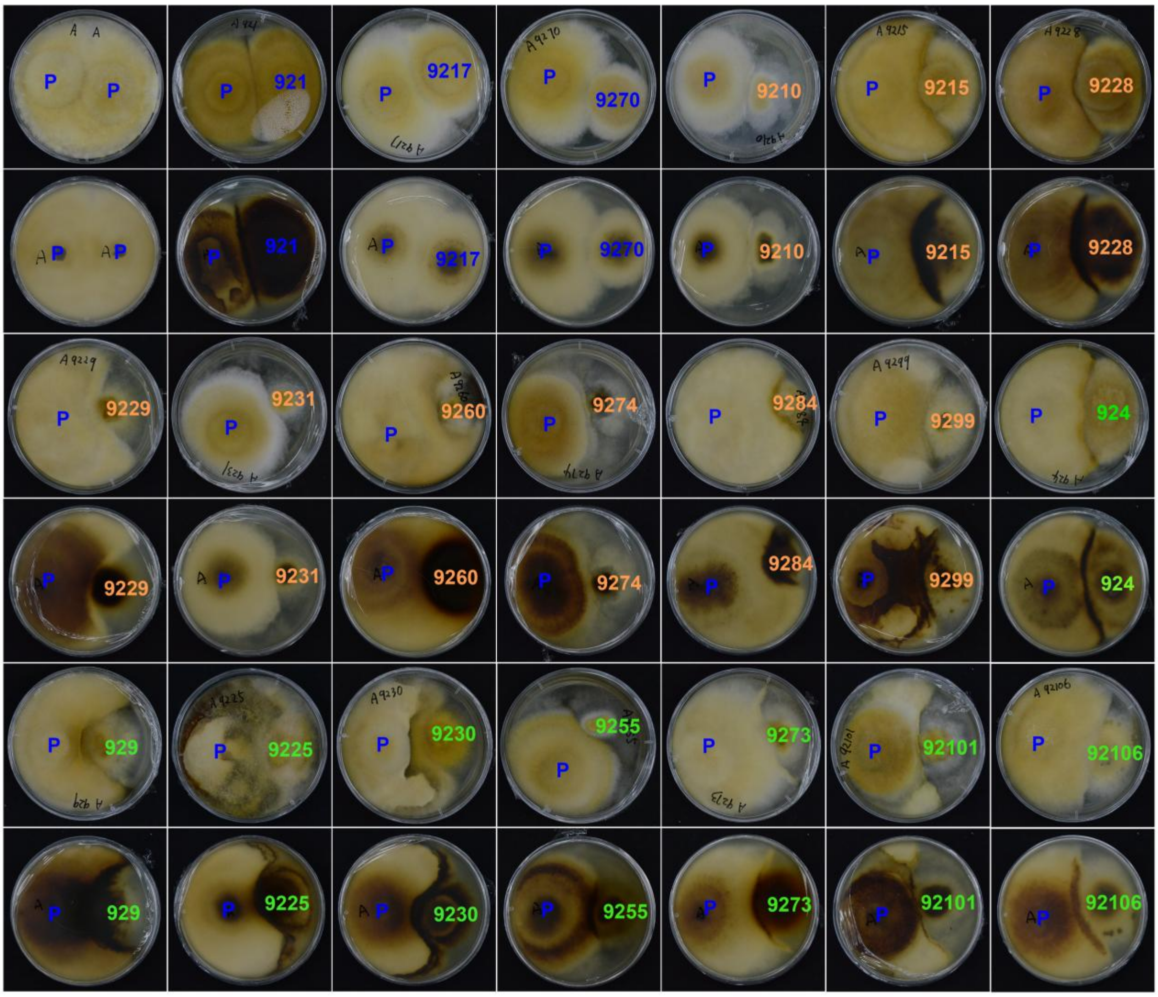

Fig. S4 Antagonistic reactions between the heterokaryotic parent strain and single spore strains. P indicates the heterokaryotic parent strain. Blue labels indicate heterokaryotic strains; orange labels indicate homokaryotic strains with mating type *A1*; green labels indicate homokaryotic strains with mating type *A2*.
